# Global burden and attributable risk factors of breast cancer in young women: historical trends from 1990 to 2019 and forecasts to 2030 by sociodemographic index regions and countries

**DOI:** 10.7189/jogh.14.04142

**Published:** 2024-07-19

**Authors:** Mengqi Yuan, Yi Zhu, Yitao Ren, Lijin Chen, Xiaochen Dai, Yuying Wang, Yixiang Huang, Hongmei Wang

**Affiliations:** 1 School of Public Health, Sun Yat-sen University, Guangzhou, Guangdong, PR China; 2 School of Health Services Management, Southern Medical University, Guangzhou, PR China; 3 Institute for Health Metrics and Evaluation, University of Washington, Seattle, Washington, USA; 4 Department of Health Metrics Sciences, School of Medicine, University of Washington, Seattle, Washington, USA; 5 Department of Radiation Oncology, Nanfang Hospital, Southern Medical University, Guangzhou, PR China

## Abstract

**Background:**

Breast cancer in young women (BCY) is much less common but has significant health sequelae and societal costs. We aimed to evaluate the global and regional burden of breast cancer in women aged 15–39 years from 1990 to 2019.

**Methods:**

We collected detailed data on breast cancer from the Global Burden of Disease Study 2019 (GBD 2019) Data Resources. The age-standardised incidence rate (ASIR), age-standardised mortality rate (ASMR), age-standardised disability-adjusted life years rate (ASDR), and estimated annual percentage change (EAPC) were used to assess the disease burden of BCY. The Bayesian Age-Period-Cohort model was used to forecast disease burden from 2020 to 2030.

**Results:**

From 1990 to 2019, significant increases in ASIR were found for BCY (EAPC = 0.59, 95% confidence interval (CI) = 0.5 to 0.68), whereas decreases in ASMR (EAPC = −0.41, 95% CI = −0.53 to −0.3) and ASDR (EAPC = −0.35, 95% CI = −0.46 to −0.24). Across countries with varying sociodemographic indexes (SDI), all regions showed an upward trend in BCY morbidity, except for countries with a high SDI. While mortality and DALYs rates have decreased in countries with high, high-middle, and middle SDI, they have increased in countries with low-middle and low SDI. Countries with lower SDIs are projected to bear the greatest burden of BCY over the next decade, including both low and low-middle categories. Alcohol use was the main risk factor attributed to BCY deaths in most countries, while exposure to second hand smoke was the predominant risk factor for BCY deaths in middle and low-middle SDI countries.

**Conclusions:**

The burden of breast cancer in young women is on the rise worldwide, and there are significant regional differences. Countries with a low-middle or low SDI face even more challenges, as they experienced a more significant and increasing BCY burden than countries with higher SDIs.

Breast cancer is the most commonly diagnosed cancer and a leading cause of death among females, with an estimated 2.3 million new breast cancer cases and 685 thousand deaths in 2020 [1]. Due to differences in the availability of early-stage screening, diagnostic procedures, and access to treatment [2,3], there are significant disparities in breast cancer mortality and morbidity between regions. For instance, the incidence rate is nearly 2.5 times higher in developed countries compared to developing regions [4]. Deaths continue to rise in low- and middle-income countries, such as those in the sub-Saharan Africa region, while the death rate is declining in high-income countries like North America [5]. Nonetheless, health care resources are extremely scarce in the low-middle income countries compared with high-income countries. The latest published participation rates in organised mammographic screening range from under 20% in Turkey, the Slovak Republic to over 80% in the USA, the Netherlands, and Finland [4]. Therefore, understanding the global and regional disease burden of breast cancer is important for allocating health care resources in different regions, especially in low-middle income countries.

The Global Burden of Diseases Study 2019 (GBD 2019) has estimated the global burden and attributable risk factors for breast cancer from 1990 to 2019 [6], and some researchers have used the GBD2019 database to predict the incidence and deaths for all ages from 2020 to 2050 [5]. However, the global burden and trend of breast cancer in young women remain unclear. Compared with older age groups, breast cancer in young women (BCY) has a significant health impact and cost to society [7]. On the one hand, BCY affects young women's sexual health and body image, which can be detrimental to their physical and mental health. On the other hand, treatment for BCY is associated with reduced fertility, which can affect individuals, families, and even society [8–10]. Given the differences in physiological and psychological factors between different age groups, identifying the risk factors related to BCY is essential for developing better interventions for young women.

Recent studies have shown that BCY is associated with several factors, including BRCA gene mutation, family history, breast density, number of births, breastfeeding, smoking, alcohol consumption, radiation, exercise, and diet [11]. Genetic and biological factors have a greater influence on BCY, but lifestyle and environmental factors such as smoking, alcohol, and physical activity are modifiable and more efficient than others. To the best of our knowledge, global or regional evidence on the modifiable risk factors for BCY is limited, and this gap may influence the effectiveness of intervention strategies at the global, regional, and national levels. GBD 2019 provides systematic estimates of the risk factors and causes of death worldwide, with stratification based on age, sex, location, and sociodemographic index (SDI), which provides an opportunity to better understand the growing burden of BCY. Sociodemographic index, developed by GBD 2019 researchers, is widely used to compare the differences in cancer burden that may be attributed to the disparity of sociodemographic development across regions [5,12,13]. For this reason, the study examined the global and regional (i.e. various SDI regions) burden of BCY and associated risk factors between 1990 and 2019 using data from the GBD 2019 database. In addition, successful and effective policymaking requires both an understanding where we are now and a prediction as to where we will be over the next decade. This can be a basis for the setting of priorities for policy implementation and the effective use of resources. In this context, we used Bayesian Age-Period-Cohort (BAPC) model to forecast the global and regional burden of BCY between 2020 and 2030. The study will provide direct evidence to inform health resource allocation and policy development related to young breast cancer globally and regionally.

## METHODS

### Data source

Estimates from the GBD 2019 study, coordinated by the Institute for Health Metrics and Evaluation, were used for the analysis of the burden of BCY and its risk factors from the years 1990 to 2019. GBD 2019 is a multinational collaborative study that estimates the diseases burden for 369 diseases and injuries and 87 risk factors across 204 countries [14,15]. As GBD 2019 estimates are based on the national registration systems, its accuracy may be associated with the quality and availability of data for each country. That is to say, there may be some gaps between the GBD 2019 estimates and reality. In order to obtain accurate and reliable estimates, the GBD collaborators use several statistical methods, including the Cause of Death Ensemble model (CODEm), spatiotemporal Gaussian process regression (ST-GPR), and the Bayesian meta-regression tool, DisMod-MR. Briefly, CODEm is a systematic tool for analysing cause of death data. It uses an ensemble of different modelling methods for rates or cause fractions, with varying choices of covariates that perform best with out-of-sample predictive validity testing. DisMod-MR is a Bayesian meta-regression tool that evaluates all available data on incidence, prevalence, remission, and mortality for a disease, ensuring consistency between epidemiological parameters. ST-GPR is a set of regression methods that borrow strength between locations and over time for single metrics of interest, such as risk factor exposure or mortality rates [16]. Previous publications provided more details on these general GBD methods [6,17].

We extracted estimates of, incidence, deaths, and disability-adjusted life-years (DALYs) across different causes, ages, all years, and locations from the GBD 2019 website (https://vizhub.healthdata.org/gbd-results/). Furthermore, as panellists of the ESO-ESMO Fifth International Consensus Guidelines on Breast Cancer in Young Women defined ‘young women’ as women under 40 years of age at breast cancer diagnosis, this study used the GBD 2019 estimates that stratified to ages 15–39 years [18]. Further details on data selection are shown in Supplementary Methods and Figure S1 in the Online Supplementary Document.

### Sociodemographic index

The SDI, developed by GBD researchers, is a summary indicator of the level of socioeconomic development in a certain country. Several studies used the indicator to compare the differences in cancer burden that may be attributed to the disparity of sociodemographic development across regions [5,12,13]. It is a composite index comprising three key indicators: the total fertility rate of persons under 25 years of age, the average educational attainment of individuals aged 15 years and over, and the lagged per capita income. The SDI is the geometric mean of the three independently estimated and scaled components, with lower values indicating lower development. The SDI ranges from 0 to 1, representing the lowest to the highest level of development, with 0 representing the fewest years of education, the lowest per capita income, and the highest fertility rate. Based on the SDI, the countries are categorised into five different SDI groups: low, low-middle, middle, high-middle, and high SDI regions [6].

### Statistical analysis

Using the 2019 data from the United Nations standard projections data set, age-standardised rates (ASR) such as age-standardised incidence rate (ASIR), age-standardised mortality rate (ASMR), and age-standardised disability-adjusted life years rate (ASDR) were computed. This data set provides population data in five-year age groups (https://population.un.org/wpp/Download/Standard/Population/) spanning from 1990 to 2030. The rates were derived based on the subsequent formula [19]:



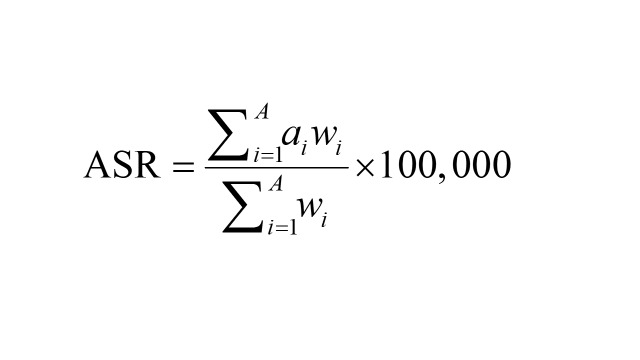



where A denotes the number of age groups, i denotes the ith age group, a_i_ is the rate to be standardised, and w_i_ is the number of standard populations in the same age.

In order to assess the changes in the burden of disease in BCY, I introduced the indicator of estimated annual percentage change (EAPC). EAPC was computed to depict the secular trend in ASR of BCY burden based on a regression model by fitting the natural logarithm of ASR with the calendar year, which is a widely employed summary measure to assess the trend of ASR within a specific time interval [19]. It is hypothesised that a linear relationship exists between the natural logarithm of ASR and time. The EAPC of ASR, along with their corresponding confidence intervals (CI), can be calculated to illustrate the temporal patterns of ASR changes from 1990 to 2019 using the following formula: EAPC = 100 × (exp(β) - 1). The EAPC is expressed on a scale of −1 to 1. An EAPC>0 indicates the increase in ASR, whereas an EAPC<0 means the decrease in ASR.

To forecast the disease burden of BCY from 2019 to 2030, we integrated global population projection data from the IHME agency (https://ghdx.healthdata.org/record/ihme-data/global-population-forecasts-2017-2100) and conducted BAPC modelling analysis using R software. The BAPC analysis serves as a primary methodology for examining the changing trends in the incidence and mortality of diseases and predicting future disease burdens [20]. According to Liu et al., we employed the BAPC model to predict ASIR, ASMR, and the number of incidences and deaths in the future decade. This model has demonstrated a better predictive performance compared to other models like the Joinpoint model and Poisson regression [21]. The BAPC model assumes a similar effect of age, period, and cohort that are adjacent in time. All unknown parameters are regarded as random with appropriate prior distributions in the BAPC model. Bayesian inference uses the second-order random walk for smoothing priors of age, period, and cohort effects. Prior knowledge combined with observed data are used to derive a posterior distribution [22]. The integrated nested Laplace approximations were used with the BAPC model to approximate the marginal posterior distributions, avoiding mixing and convergence issues introduced by Markov chain Monte Carlo sampling techniques traditionally used in the Bayesian approach [23]. By incorporating both sample data and prior information, the model ensures distinct parameter estimates, thereby guaranteeing consistent and dependable outcomes. All data analyses were conducted using the open-source software R (version 4.2.1; R Foundation for Statistical Computing, Vienna, Austria).

The study was based on a publicly available data set. Each step used to analyse the GBD database in the current study followed the guidelines of cross-sectional study described in the Guidelines for Accurate and Transparent Health Estimates Reporting (GATHER) [24]. All R code supporting the conclusions of this study can be accessed and downloaded via Github (https://github.com/Mrbai77/Code-of-GBD-for-BCY).

## RESULTS

### Global burden and trend of breast cancer in young women

In 2019, the global age-standardised incidence rate of BCY per 100 000 population was 11.54 (95% uncertainty interval (UI) = 10.4 to 12.75) (**Table 1**). The country with the highest BCY incidence was 19 times higher than the country with the lowest incidence. Solomon Islands (ASIR = 55.19, 95% UI = 31.94 to 85.34), Monaco (ASIR = 41.99, 95% UI = 25.76 to 66.56), and Lebanon (ASIR = 35.72, 95% UI = 22.14 to 54.5) were the three countries with the highest incidence rate per 100 000 of young female breast cancer, whereas Gambia (ASIR = 2.92, 95% UI = 1.65 to 4.77), Niger (ASIR = 2.92, 95% UI = 1.49 to 5.28), and Somalia (ASIR = 3.07, 95% UI = 1.38 to 5.41) had the lowest ASIR in 2019 (Table S1 and Figure S2 in the **Online Supplementary Document**). From 1990 to 2019, the global incidence of breast cancer in young women increased from 89 174.14 to 168 775.84, and the number of cases increased by 89.27% (Table S2 in the **Online Supplementary Document**). Among 204 countries, the Solomon Islands (EAPC = 8.25, 95% CI = 7.41 to 9.09) and Saudi Arabia (EAPC = 4.02, 95% CI = 3.86 to 4.18) presented the highest average annual increase, whereas Saint Kitts and Nevis (EAPC = −3.98, 95% CI = −4.41 to −3.55) showed the highest average annual decrease (Table S3 and Figure S3 in the **Online Supplementary Document**).

**Table 1:** The global and five SDI region disease burden of breast cancer in young women

Measure/region	2019, number, No. (95% UI)	2019, ASR per 100 000, No. (95% UI)	1990–2019, EAPC, No. (95% CI)
**Incidence**			
Global	168 775.84 (153 043.02 to 185 086.12)	11.54 (10.4 to 12.75)	0.59 (0.5 to 0.68)
High SDI	31 364.78 (27 869.73 to 35 260.3)	17.47 (15.38 to 19.73)	−0.19 (−0.25 to −0.12)
High-middle SDI	39 210.95 (34 230.85 to 44 944.22)	13.65 (11.86 to 15.77)	0.92 (0.8 to 1.03)
Middle SDI	55 109.03 (48 311.56 to 62 105.16)	11.53 (10.09 to 13.12)	1.57 (1.46 to 1.68)
Low-middle SDI	30 005.1 (25 926.72 to 34 333.2)	8.86 (7.47 to 10.51)	1.23 (1.1 to 1.36)
Low SDI	12 956.23 (10 821.88 to 15 238.12)	6.9 (5.65 to 8.26)	1.35 (1.28 to 1.42)
**Deaths**			
Global	42 742.36 (38 756.19 to 46 959.81)	2.92 (2.64 to 3.24)	−0.41 (−0.53 to −0.3)
High SDI	3744.77 (3569.64 to 3929.61)	2.07 (1.95 to 2.19)	−1.81 (−1.94 to −1.67)
High-middle SDI	6751.45 (6095.32 to 7494.5)	2.34 (2.09 to 2.61)	−1.21 (−1.37 to −1.05)
Middle SDI	14 016.94 (12 400.21 to 15 782.02)	2.93 (2.59 to 3.32)	−0.25 (−0.35 to −0.14)
Low-middle SDI	11 853.32 (10 080.2 to 13 865.28)	3.52 (2.93 to 4.21)	0.1 (−0.03 to 0.24)
Low SDI	6334.04 (5334.35 to 7495.81)	3.43 (2.84 to 4.09)	0.51 (0.44 to 0.57)
**DALYs**			
Global	2 468 523.41 (2 238 022.2 to 2 701 471.24)	168.84 (151.85 to 186.22)	−0.35 (−0.46 to −0.24)
High SDI	225 341.22 (212 350.5 to 239 871.44)	125.19 (117.09 to 134.02)	−1.65 (−1.78 to −1.52)
High-middle SDI	393 755.8 (355 560.5 to 433 631.52)	136.87 (122.81 to 152.02)	−1.09 (−1.24 to −0.93)
Middle SDI	804 877.24 (713 563.17 to 901 075.71)	168.48 (148.57 to 189.84)	−0.17 (−0.28 to −0.07)
Low-middle SDI	681 198.7 (580 834.92 to 794 381.78)	201.27 (166.66 to 240.97)	0.15 (0.02 to 0.28)
Low SDI	360 974.39 (303 731.97 to 426 345.4)	193.02 (159.84 to 230.83)	0.54 (0.47 to 0.6)

ASR – age-standardised rate, CI – confidence intervals, DALYs – disability-adjusted life-years, EAPC – estimated annual percentage change, SDI – sociodemographic index, UI – uncertainty interval

In 2019, the global age-standardised mortality rate and DALYs rate of BCY per 100 000 population were 2.92 (95% UI = 2.64 to 3.24) and 168.84 (95% UI = 151.85 to 186.22), respectively (**Table 1**). Mortality rates for BCY in countries with the highest rates were 20 times higher than those in countries with the lowest rates. Solomon Islands (ASMR = 23.42, 95% UI = 13.88 to 35.64), Papua New Guinea (ASMR = 11.91, 95% UI = 7.47 to 18.21), and Pakistan (ASMR = 9.76, 95% UI = 6.44 to 14.41) were the three countries with the highest mortality rates per 100 000 of BCY, whereas Singapore (ASMR = 1.13, 95% UI = 0.86 to 1.45), Kuwait (ASMR = 1.25, 95% UI = 0.85 to 1.82), and Honduras (ASMR = 1.4, 95% UI = 0.75 to 2.38) had the lowest ASMR in 2019 (Table S1 and Figure S4 in the **Online Supplementary Document**). From 1990 to 2019, the global mortality cases of BCY increased from 29 759.35 to 42 742.36, and the number of cases increased by 43.63% (Table S2 in the **Online Supplementary Document**). Among 204 countries, the Solomon Islands (EAPC = 7.57, 95% CI = 6.69 to 8.45) and Zimbabwe (EAPC = 3.57, 95% CI = 2.57 to 4.41) had the highest average annual increase, while Saint Kitts and Nevis (EAPC = −5.17, 95% CI = −5.68 to −4.67) had the highest average annual decrease (Table S3 and Figure S5 in the **Online Supplementary Document**). ASDR show similar trends with ASMR in different countries (Tables S1 and S3, Figures S6–7 in the **Online Supplementary Document**).

### Regional burden and trend of breast cancer in young women

**Figure 1:**
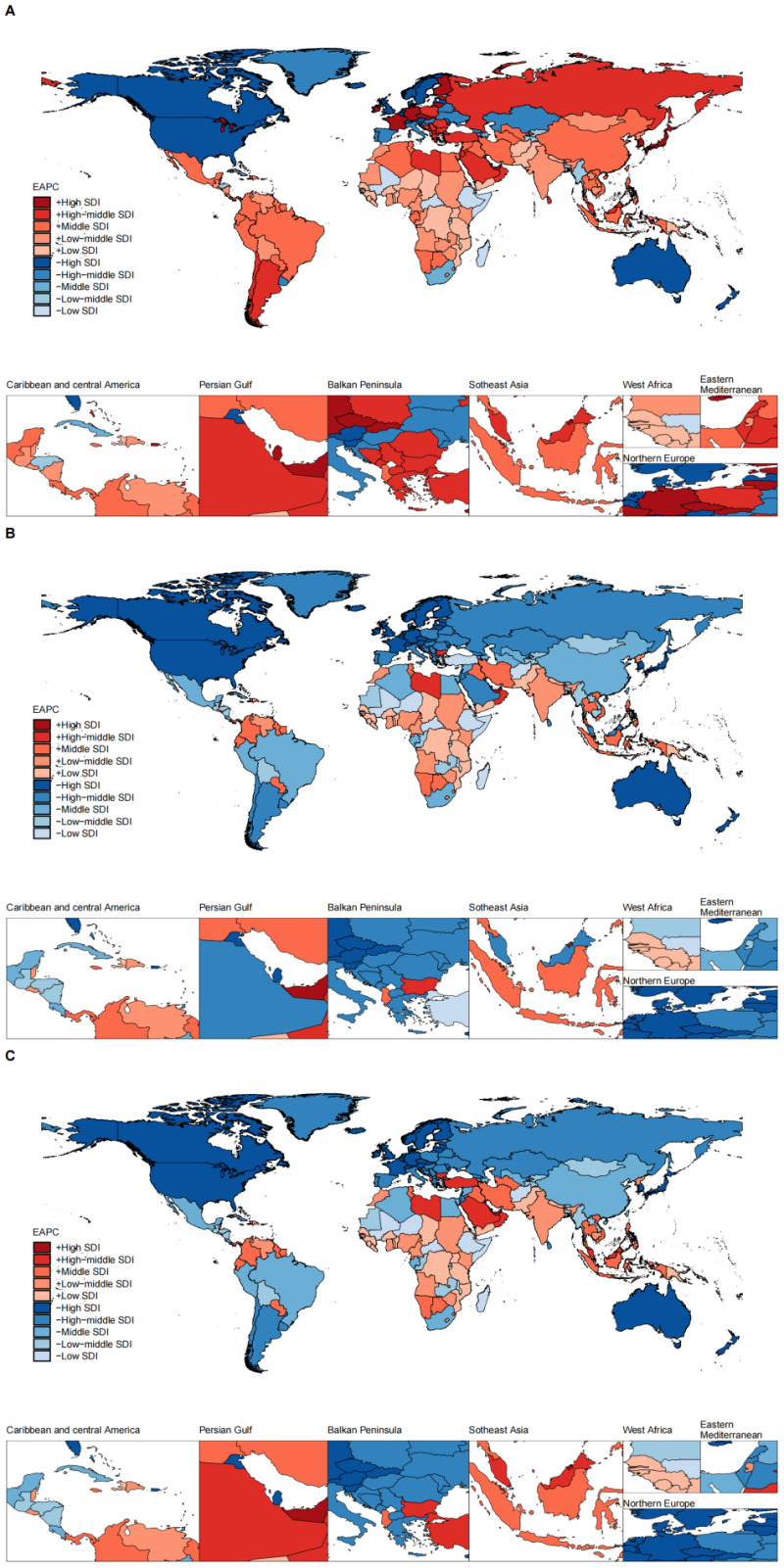
Estimated annual percentage change in the global burden of disease for breast cancer in young women in 204 countries and territories between 1990 and 2019. **Panel A.** The EAPC in ASIR. **Panel B.** The EAPC in ASMR. **Panel C.** The EAPC in ASDR. Red indicates that the EAPC is greater than zero, blue indicates that the EAPC is less than zero, and different colour differences indicate that the country belongs to a different SDI level. ASIR – age-standardised incidence rate, ASDR – age-standardised disability-adjusted life years rate, ASMR – age-standardised mortality rate, EAPC – estimated annual percentage change

Throughout the study, regardless of the level of SDI, there were countries in which the EAPC for ASIR, ASMR, and ASDR was greater than zero, meaning these three indicators were on an upward trend from 1990–2019 (**Figure 1**, panels A–C). **Table 1** shows the global burden of BCY across different regions in 2019. Based on the socio-demographic index, countries with a high SDI had the highest ASIR of BCY per 100 000 population (ASIR = 17.47, 95% UI = 15.38 to 19.73), while countries with a low SDI had the lowest ASIR (ASIR = 6.9, 95% UI = 5.65 to 8.26), a difference of 2.5 times. The data show a positive correlation between the SDI level and the ASIR. From 1990 to 2019, the high SDI region was the only category to show a downward trend in ASIR (EAPC = −0.19, 95% UI = −0.25 to −0.12), while other SDI regions showed an upward trend, with an EAPC>0.

In 2019, high SDI regions had the lowest rates of ASMR (ASMR = 2.07, 95% UI = 1.95 to 2.19) and ASDR (ASDR = 125.19, 95% UI = 117.09 to 134.02) among the five groups. For the trend of ASMR and ASDR, lower SDI regions (including low and low-middle SDI regions) had the highest rates and showed an increasing trend (EACP>0), and both higher SDI regions (including high and high-middle SDI regions) and middle SDI regions showed a decreasing trend (EACP<0) (**Table 1**).

### Projected future global burden of young women with breast cancer

**Figure 2:**
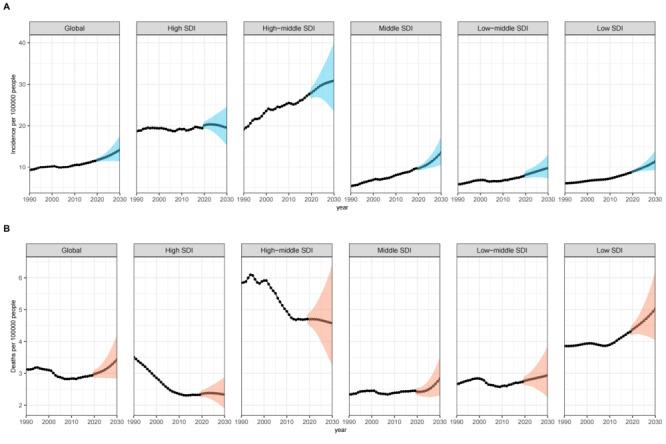
Incidence and deaths for young female breast cancer projections at the global and five SDI levels for 15–39 years-old, 1990–2030. **Panel A.** ASIR (per 100 000) by global and SDI region from 1990 to 2030. **Panel B.** ASMR (per 100 000) by global and SDI region from 1990 to 2030. The solid point represents the observed values, and the predictive value is shown as a hollow point. The shaded regions represent the 95% confidence intervals. ASIR – age-standardised incidence rate, ASMR – age-standardised mortality rate, SDI – social development index

The BAPC model predicts an increasing disease burden associated with BCY over the next 10 years. The ASMR and ASIR show a gradual upward trend worldwide (**Figure 2**, Table S4 in the **Online Supplementary Document**).

**Figure 2** presents the projections of the global disease burden in BCY across different regions. There is an overall increasing trend in the ASIR across five SDI quintiles. Specifically, the high-middle SDI quintile has the highest ASIR. The predicted data show that only the high SDI areas are seeing a decrease in incidence, whereas all other regions are on an upward trend (**Figure 2**, panel A). For ASMR, the projections showed a decrease in the high and high-middle SDI quintiles, while an increase was observed in the remaining SDI quintiles (**Figure 2**, panel B). According to the results between 2020 and 2030, the most significant increases in ASIR and ASMR were observed in the middle SDI regions, with rises of 40.14 and 16.94%, respectively. Simultaneously, it is noteworthy that ASIR increases within the high-middle SDI regions, while ASDR shows a decreasing trend (Table S4 in the **Online Supplementary Document**).

### Risk factors attributable to the burdens of breast cancer in young women

**Figure 3:**
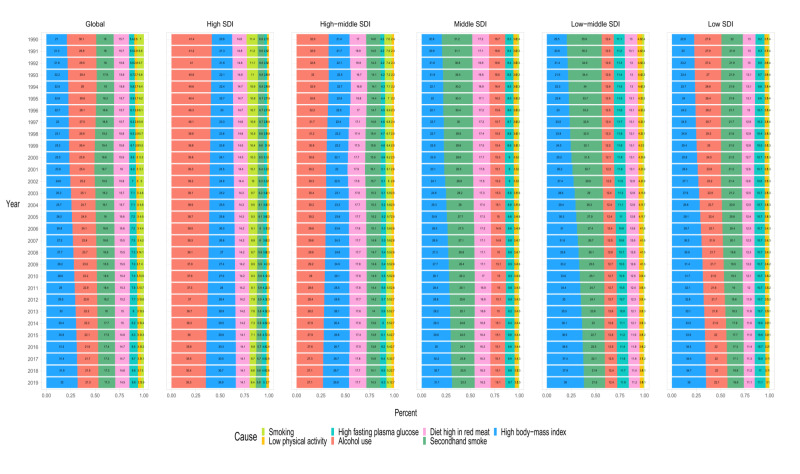
The GBD of breast cancer deaths in young women is attributable to factors in different SDI regions and years. GBD – Global Burden of Disease, SDI – sociodemographic index

As shown in **Figure 3**, alcohol use, second hand smoke, a diet high in red meat, high fasting plasma glucose, low physical activity, smoking, and a high body mass index (BMI) were identified as the main determinants of BCY mortality worldwide from 1990 to 2019. On a global scale, alcohol use emerged as the most important risk factor among BCY patients, accounting for 30.1% of cases in 1990 and gradually decreasing to 21.3% by 2019. Secondhand smoke emerged as the second most important risk factor, showing a consistent pattern over the last three decades. Intriguingly, the beneficial impact of a high BMI as a protective factor showed a significant upward trend.

Furthermore, the contributions of different risk factors varied by region. High BMI was identified as a protective factor for BCY across all areas. Alcohol use emerged as the main risk factor for BCY mortality in regions characterised by higher SDIs (including high and high-middle SDI regions) and in regions with low SDI. In the middle and low-middle SDI regions, secondhand smoke emerged as the primary risk factor. The percentages of BCY deaths due to both alcohol use and secondhand smoke showed a gradual downward trend, which means these two risk factors have a decreasing influence among all influences. The decrease in BCY deaths due to alcohol consumption was most pronounced in areas with the high SDI region, from 41.4 in 1990 to 35.3% in 2019. In the middle and low-middle SDI regions, BCY mortality associated with secondhand smoke decreased by 25.32 and 39.27%, respectively. In regions with high and high-middle SDI, low physical activity was the least impactful risk factor. On the other hand, smoking was identified as the least significant risk factor in middle and lower SDI regions, which included both low and low-middle SDI regions.

## DISCUSSION

The incidence of breast cancer in young women has increased significantly, although global mortality and DALYs from BCY have decreased between 1990 and 2019. Over the next decade, there will be an increase in global breast cancer incidence and mortality rates in young women. Furthermore, we observed that the burden and patterns of attributable risk factors for BCY varied across five SDI groups.

Consistent with previous studies focusing on the total population (aged 15–90), we found that all countries, except the high SDI region, are showing an increasing trend in breast cancer incidence among young women. Widespread implementation of screening is a major contributor to the increasing trend of breast cancer [25]. Since the twentieth century, countries such as the USA, the UK, Australia, and Canada have implemented large-scale breast cancer screening programmes. Countries such as Mexico, Vietnam and South Africa have also implemented small screening programmes [26,27]. Existing research suggests that routine screening is not beneficial in the younger age group and that an individualised screening strategy is needed; thus, free screening programmes in most countries are mainly targeting middle-aged and elderly women [28,29]. However, mass screening, the Pink Ribbon Breast Cancer Awareness Campaign of 1992 and other similar activities have raised awareness of the fight against breast cancer and encouraged young women at risk to seek screening [30]. Studies generally agree that magnetic resonance imaging is an appropriate screening method for young women at high risk of breast cancer, but it is more expensive than other screening methods such as clinical breast examination, mammography and breast ultrasonography, making it unaffordable for people in low-income countries [31–33]. Even if it is subsidised by the government, it can still be a significant financial burden. This may be one of the main reasons for BCY with the low incidence rates in middle, low-middle, and low SDI countries. In most countries with a high SDI, the incidence of BCY has been declining. Increased awareness of breast cancer and early mass screening led to more precancerous lesions detected may contributing to the decline in the incidence rate [7].

Our study also found an inverse association between the SDI and the age-standardised DALY and mortality rate. However, results for all ages showed countries with a middle SDI had the lowest mortality of breast cancer [34]. The disparity may be due to differences in age-specific screening programmes, malignancy of the disease, and levels of medical intervention between countries [35]. Most countries with high SDI, such as North America and Europe, advocate for biennial mammography screenings beginning at the age of 50, but some younger women may get a mammography before that age [36]. Compared with high SDI countries, the screening of breast cancer is not yet widely implemented in low SDI regions, such as sub-Saharan Africa [37]. Inadequate screening could contribute to higher mortality in these countries, as the delayed diagnosis of breast cancer is associated with adverse outcomes. Additionally, BCY exhibits greater malignancy compared to that in older patients. It posed challenges in treatment due to its aggressive nature and demanded more extensive medical resources, which were often more readily available in developed nations [38,39]. Taken together, it is reasonable that the observed breast cancer mortality in young women was lowest in the high SDI region. The lower mortality rate observed in the all-age group in the middle SDI region may be the result of a relatively high level of treatment combined with a lower level of screening [13].

The decline in BCY mortality in high-resource countries from 1990 to 2019 can be attributed to technological advances and better understanding of disease biology. These include the introduction of chemotherapy in the 1970s, breast-conserving surgery and the widespread use of tamoxifen in the 1980s, sentinel node biopsy in the 1990s, and improved imaging techniques and models of care for young adults with cancer [40,41]. However, more advanced treatments and medical system often come with higher costs, which are unaffordable for a significant portion of the population and national financial systems in low and low-middle SDI areas. Examples include the UK's national network of young adult cancer units, the US Affordable Care Act, numerous breast cancer-related laws, the elevated costs of treatments like trastuzumab, pertuzumab, three-dimentional digital breast tomosynthesis and other therapeutic drugs used in high-income countries [42,43]. In developing countries where resources are scarce, doctors may lack the expertise to utilise advanced instruments and high-quality imaging equipment may be unavailable. Additionally, targeted receptor therapies, such as trastuzumab, remain inaccessible to many women in sub-Saharan African countries due to their high cost [44,45]. The study revealed that some countries were not following the expected trend based on their SDI region. For instance, Zambia, a low-middle SDI country in Africa, has a decreasing mortality rate. Zambia has developed its breast cancer control programme by building on its existing cervical cancer screening and treatment programme. Trained cervical cancer screening nurses at primary health clinics perform clinical breast examinations and refer women with possible abnormalities. This approach necessitates a minimal new infrastructure or additional human costs [46].

Further projections show that incidence and mortality would increase annually for the burden of BCY. Regionally, ASIR is decreasing in countries with high SDI, but areas with lower SDI, including low, low-middle and middle SDI, tend to increase in ASIR over the next decade. The findings suggest that, in the absence of effective control and prevention measures, the socio-economic and health systems of countries with lower SDIs will face enormous challenges. For example, the loss of young fertility may exacerbate the ageing of the population, leading to more social conflicts and burdens. Young women constitute a vital segment of the labour force. Consequently, labour loss associated with breast cancer has a significant impact on the functioning of society. In this context, it is very important to take appropriate measures to address the challenges. A cost-effectiveness analysis of breast cancer control strategies in Ghana found that biennial clinical breast examination was the most cost-effective intervention, and the incremental cost per DALYs saved was around 10 times lower than mammography screening, which is very meaningful for low and low-middle SDI countries [47]. Significantly, policymakers should consider the country-specific development status and the characteristics of the death burden in the SDI regions it belongs to when developing and implementing strategies to prevent and reduce the disease burden generated by BCY. Increased funding for breast cancer screening and individualised BCY screening will place a greater burden on the country's financial system. At the same time, in certain regions, delays in care seeking can be attributed to cultural influences, fatalism, fear of stigma, a preference for folk prescription, fear of a positive diagnosis, fear of pain and cost, and the perception that treatments such as mastectomies may cause feelings of worthlessness in some women [44,48]. Therefore, screening promotion in resource-poor areas should not be based solely on Western criteria, but also on local realities. Raising awareness of breast cancer prevention and providing individualised screening for high-risk groups is crucial for specific groups of young women to optimise the use of limited resources. As an important part of a holistic approach to breast cancer management, genetic screening should be offered to high-risk young women where resources allow [49]. Our findings provide direction for future research endeavours. For instance, further research should examine the effectiveness of early screening programmes in reducing mortality in regions with low SDI or investigate the influence of cultural factors on preventive measures for BCY, among other possibilities.

Six risk factors were associated with the global mortality of young breast cancer, including alcohol use, secondhand smoke, a diet high in red meat, high fasting plasma glucose, low physical activity, and smoking. Previous studies found that alcohol use was the most prominent risk factor for BCY [50]. Alcohol is the most commonly abused substance among young females. The adolescent period is critical to breast development and is, therefore, more vulnerable to the detrimental effects of carcinogens [51]. Specifically, alcohol exposure in young females has raised estradiol and progesterone levels in the bloodstream. It is advisable to consider additional support and brief interventions targeting alcohol use during the clinical trajectory [52,53]. Using the GBD database, our study identified alcohol use and secondhand smoke as important risk factors for BCY. Individuals exposed to high levels of passive smoking are shown to have an elevated risk of developing breast cancer. Specifically, the risk increases by over 30% for those exposed to passive smoking for more than 10 years throughout childhood or maturity in a workplace setting or for more than 20 years during adulthood in a home environment [54]. Additionally, excessive consumption of red meat has been linked to an elevated risk of BCY [55]. Therefore, the implementation of smoking cessation policies to minimise secondhand smoke exposure, along with the adoption of a nutritious contemporary dietary pattern, may contribute to the prevention of BCY among individuals residing in communal environments [55,56]. In addition, the findings presented in this study diverge from the majority of research endeavours that have explored the burden of breast cancer. The study showed that a high BMI acted as a protective factor for BCY. This association may be due to the reduced ovarian hormone production associated with increased adiposity, which lowers the risk of breast cancer [57].

The degree of contribution from these seven factors varied across different regions. The leading risk factors associated with higher SDIs (including high and high-middle SDI regions) were alcohol use and a diet high in red meat. However, the primary risk factors in lower SDIs areas (including low and low-middle SDI regions) were exposure to secondhand smoke and alcohol use. The issue of secondhand smoke exposure warrants special attention in areas with lower SDI rankings. This stems from the link between socio-economic standing and tobacco consumption, where higher tobacco usage is often seen in populations with lesser income and educational attainment [58]. Simultaneously, it was plausible that the percentage of risk factors associated with exposure to secondhand smoke was higher in lower SDI areas due to the limited efficacy of public smoking bans in these regions. This is further compounded by a higher proportion of male smokers compared to females and a greater level of passive acceptance of secondhand smoke among young females. One of the primary risk factors observed in higher SDIs areas, but not in lower SDIs areas, is a diet characterised by a high consumption of red meat. This discrepancy can be attributed to variations in dietary patterns across different SDIs and economic contexts within the regions. Substantial amount of meat consumption is found in high SDI nations like the USA and Europe. In contrast, in middle and low-middle SDI countries such as Asia, cereals and vegetables play a key role in the dietary habits of people, with comparatively lower levels of meat or meat product consumption [59]. Research has indicated a positive correlation between a nation’s affluence and the per capita consumption of meat [60]. The correlation between increased affluence and higher meat eating implies that developing countries should place red meat safeguards and adopt healthier dietary practices as economies progress. Meanwhile, it is necessary to emphasise prevention strategies in various SDI regions. Specifically, heightened regulation of alcohol consumption should be implemented in high, high-middle, and low SDI areas. At the same time, increased safeguards against secondhand smoke exposure should be established in middle and low-middle SDI locations. Several initiatives, such as those targeting tobacco control, have demonstrated effectiveness in regions with higher SDI scores [61]. These interventions should be modified and implemented in nations with lower to middle SDI scores. Therefore, these countries should improve and strictly enforce laws and regulations prohibiting the sale of cigarettes and alcohol to young population, and actively promote the implementation of laws such as banning smoking in public places. In addition, it is crucial to conduct public awareness campaigns, such as breast cancer awareness campaigns, to raise awareness of risk factors so that people can take informed precautions.

To our knowledge, this study is the first to describe the global burden and trend of BCY in incidence, mortality, and DALYs lost from 1990 to 2019. It emphasises the importance of enhancing tertiary prevention strategies, raising awareness of breast health, particularly in low and low-middle SDI areas, and providing more effective individualised screening for young women. The findings, as mentioned above, can enhance our understanding of the magnitude of BCY, thereby facilitating the rational advancement of BCY prevention and treatment strategies, as well as the equitable allocation of health care resources. The study has several limitations that should be acknowledged. First, the most recent publicly available data in the GBD 2019 database are up to 2019, and it lacks burden of disease data for more recent years, which may limit the timeliness of the findings. The data used in this study come from the GBD project, which integrates epidemiological survey data and applies rigorous statistical methods to adjust for missing data. The lack of raw data for many countries means that differences between countries and regions are made up of both true differences in burden and differences due to uncertainty in estimates because of lack of data [62,63]. Additionally, the GBD database does not encompass detailed clinical information, therapeutic interventions, or influences such as genetics related explicitly related to early-onset breast cancer, which limits the depth of understanding regarding the underlying causes of the observed disparities. Finally, our projections do not take into account the potential impact of the pandemic. With the ongoing COVID-19 pandemic, delays and disruptions in cancer screening, diagnosis, and treatment worldwide may change the epidemiological pattern of BCY, resulting in a trend towards lower incidence but increased mortality [64,65].

## CONCLUSIONS

Although the age-standardised mortality rate and DALYs due to BCY have decreased worldwide over the past three decades, the number of young women diagnosed with breast cancer has continued to increase. Countries with a low-middle or low SDI face even more severe health care challenges, as they have a larger burden and growing trend of young breast cancer than countries with higher SDIs. Emphasising the importance of early individualised screening for BCY, improving the quality of clinical diagnosis and treatment, promoting healthy lifestyles, and reducing exposure to carcinogens are potential strategies to help mitigate the impact of BCY on a global scale.

## Additional material


Online Supplementary Document
