# Supplementary material for: Global burden and attributable risk factors of breast cancer in young women: historical trends from 1990 to 2019 and forecasts to 2030 by sociodemographic index regions and countries
Source: J Glob Health. 2024 Jul 19;14:04142. doi: 10.7189/jogh.14.04142 (PMC11258534; doi:10.7189/jogh.14.04142)
Supplement: Online Supplementary Document [file jogh-14-04142-s001.pdf]

Supplementary Materials Legend

Supplementary Texts

Supplementary Methods: Detailed process description for extracting data from the GBD 2019 database.

Supplementary Figures

Figure S1: Flowchart of data extraction for this study.

Figure S2: Age-standardised incidence rates of breast cancer in young women in 204 countries and territories, in 2019.

Figure S3: Estimated annual percentage change of age-standardised incidence rates of breast cancer in young women in 204 countries and territories, between 1990 and 2019.

Figure S4: Age-standardised mortality rates of breast cancer in young women in 204 countries and territories, in 2019.

Figure S5: Estimated annual percentage change of age-standardised mortality rates of breast cancer in young women in 204 countries and territories, between 1990 and 2019.

Figure S6: Age-standardised dalys rates of breast cancer in young women in 204 countries and territories, in 2019.

Figure S7: Estimated annual percentage change of age-standardised dalys rates of breast cancer in young women in 204 countries and territories, between 1990 and 2019.

Figure S8: All-age incidence and mortality numbers and rates for female breast cancer patients, 1990-2030.

Supplementary Tables

Table S1: ASIR, ASMR, ASDR of breast cancer in young women among 204 countries in 2019.

Table S2: Global Burden of Disease in Young Women with Breast Cancer by Age Group 15-39 Years.

Table S3: Estimated annual percentage change of ASIR, ASMR, and ASDR for breast cancer in young women in 204 countries.

Table S4: The global and five SDI area disease burden for young breast cancer incidence and mortality rates and changes in 2020-2030.

Supplementary Methods: Detailed process description for extracting data from the GBD 2019 database.

To extract the raw data, we need to follow a process. First, click on the website above to enter the data retrieval interface, where different GBD evaluation options can be selected from the drop-down menu in "GBD Estiate". By default, " Cause of death or injury" is selected, in which specific options can be chosen according to the content of your study. Next, we can select various disease assessment metrics in the "Measure" menu bar, such as Deaths, DALYs, Prevalence, Incidence, etc. Then in the "Metric" drop-down menu, we can select different measures, such as Number, Percent, Rate. Then select the various diseases to be studied in the "Cause" drop-down menu, e.g. lung cancer, malaria, diabetes, etc. In the "Location" drop-down menu, select the countries and regions to be included in the study, such as the United States, the United Kingdom, China, five SDI countries, the World Bank region, and so on. However, most countries do not have access to domestic data at the provincial or state level, with the exception of a few countries such as the United States, the United Kingdom, and Japan. Similarly, in the "Age" and "Sex" menus, we can select the age and gender required for the study, the age being most subdivided into intervals of 5 years. In the "Year" menu, select any year between 1990 and 2019. Because GBD 2019 data are open source, after selecting the type of data needed for the study, click "Search" to retrieve the information, or you can choose to download the CSV file directly by clicking the "Download CSV" button.

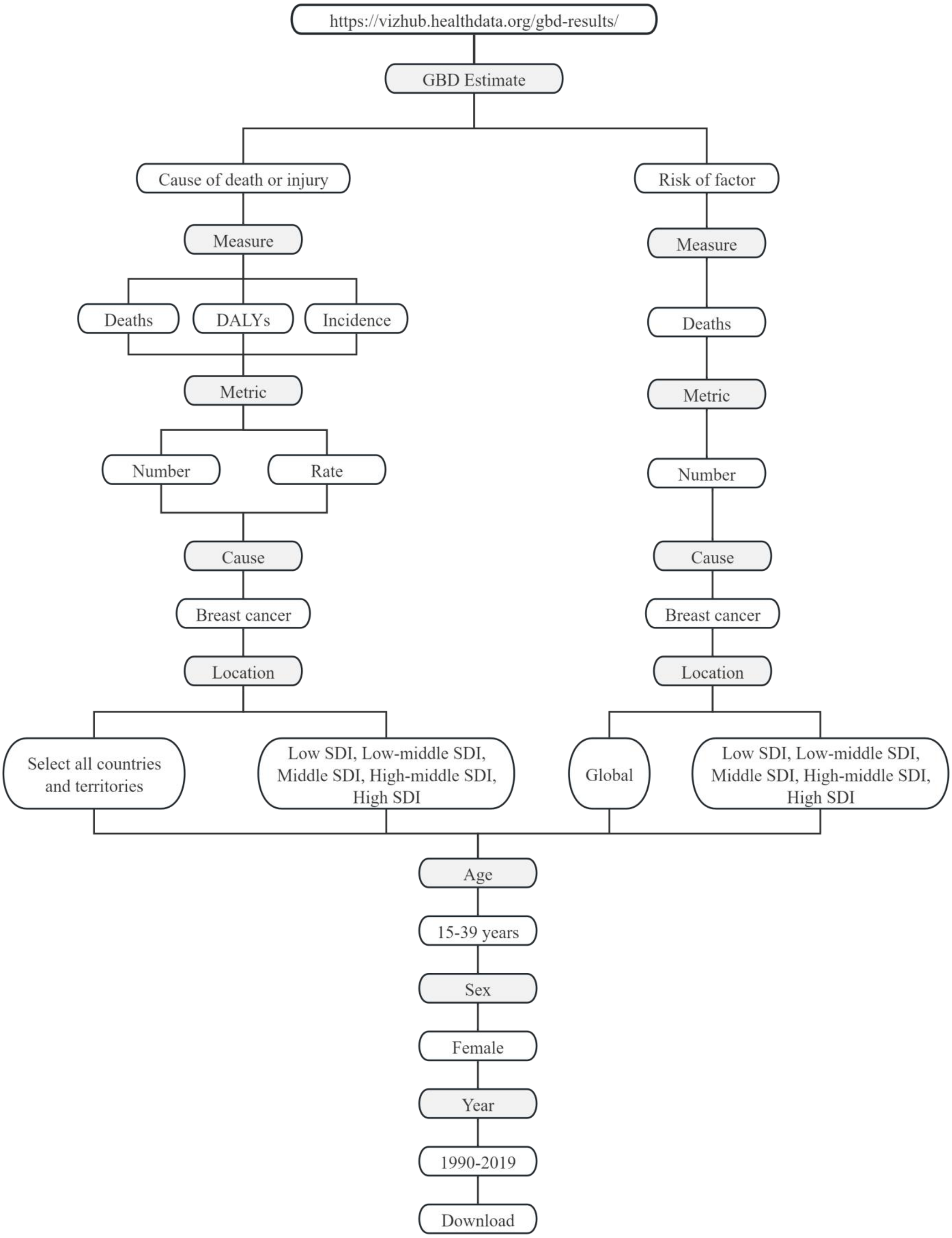

Figure S1: Flowchart of data extraction for this study.

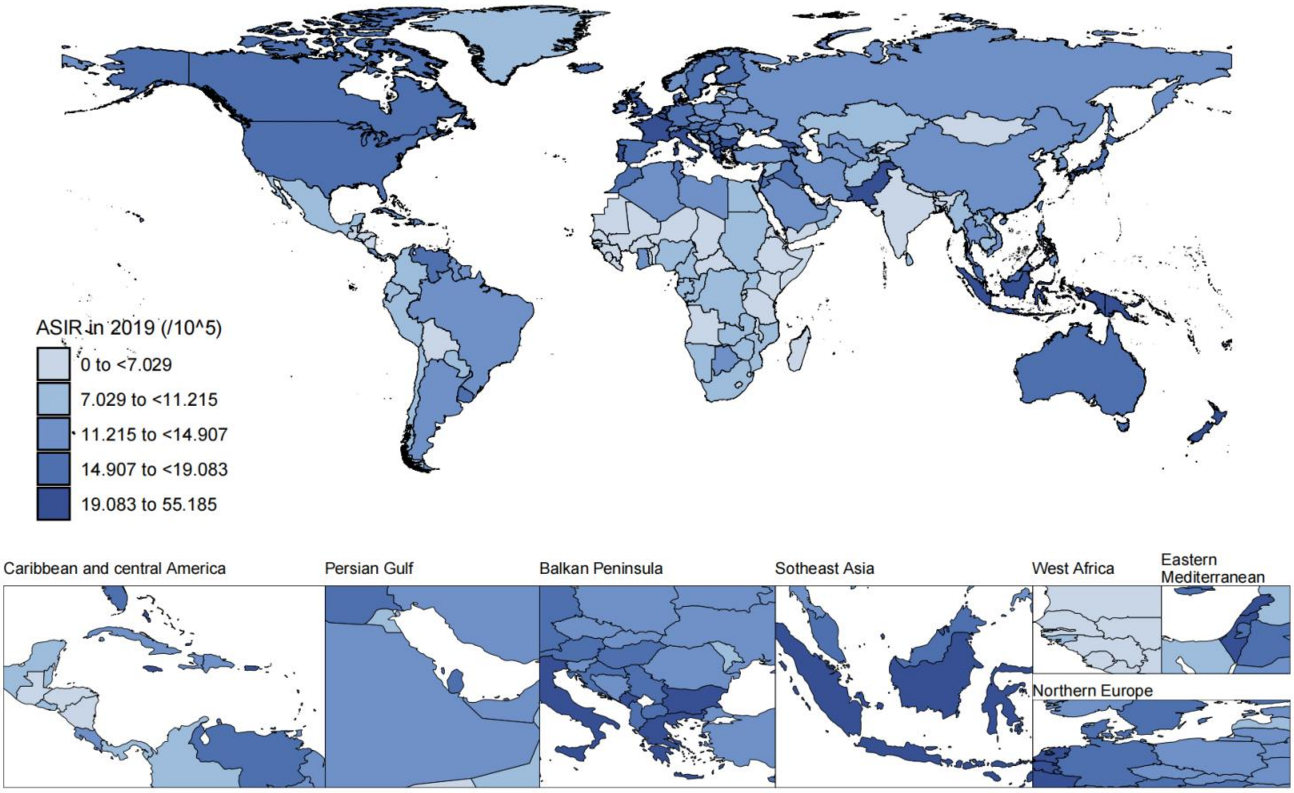

Figure S2: Age-standardised incidence rates of breast cancer in young women in 204 countries and territories, in 2019.

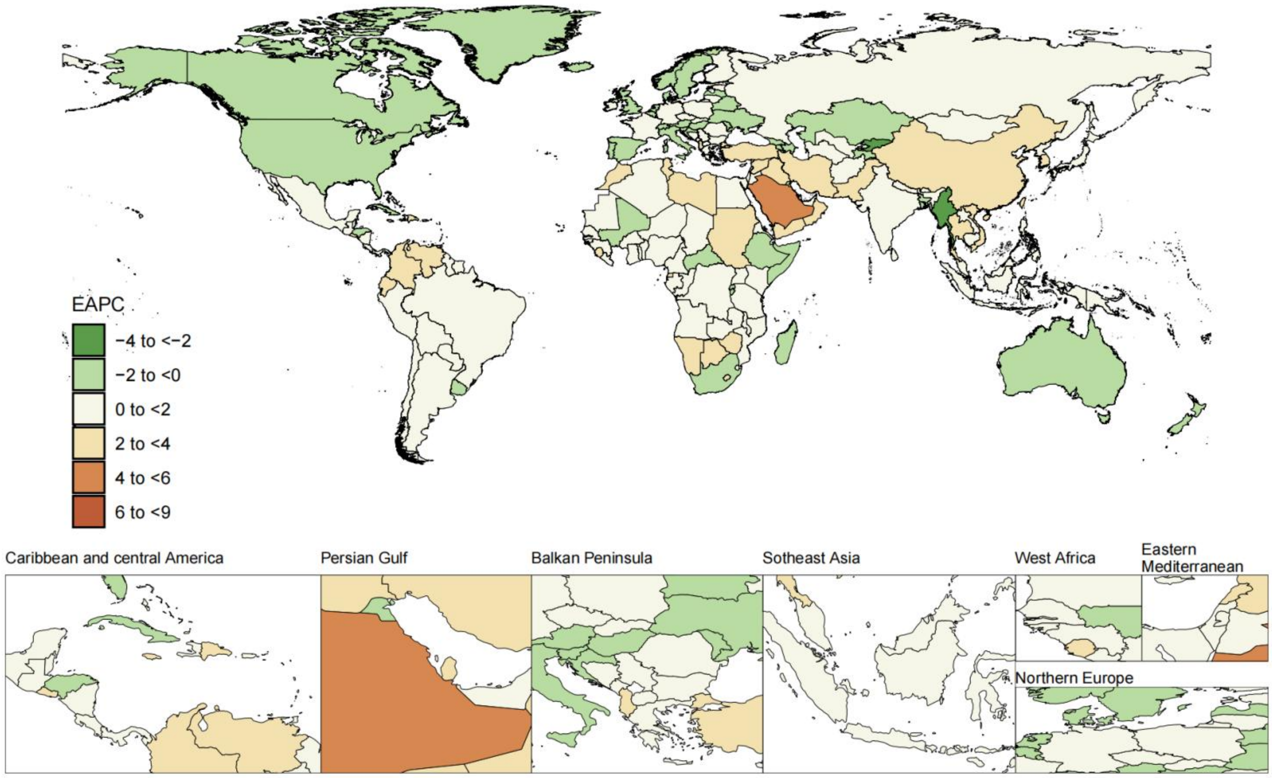

Figure S3: Estimated annual percentage change of age-standardised incidence rates of breast cancer in young women in 204 countries and territories, between 1990 and 2019.

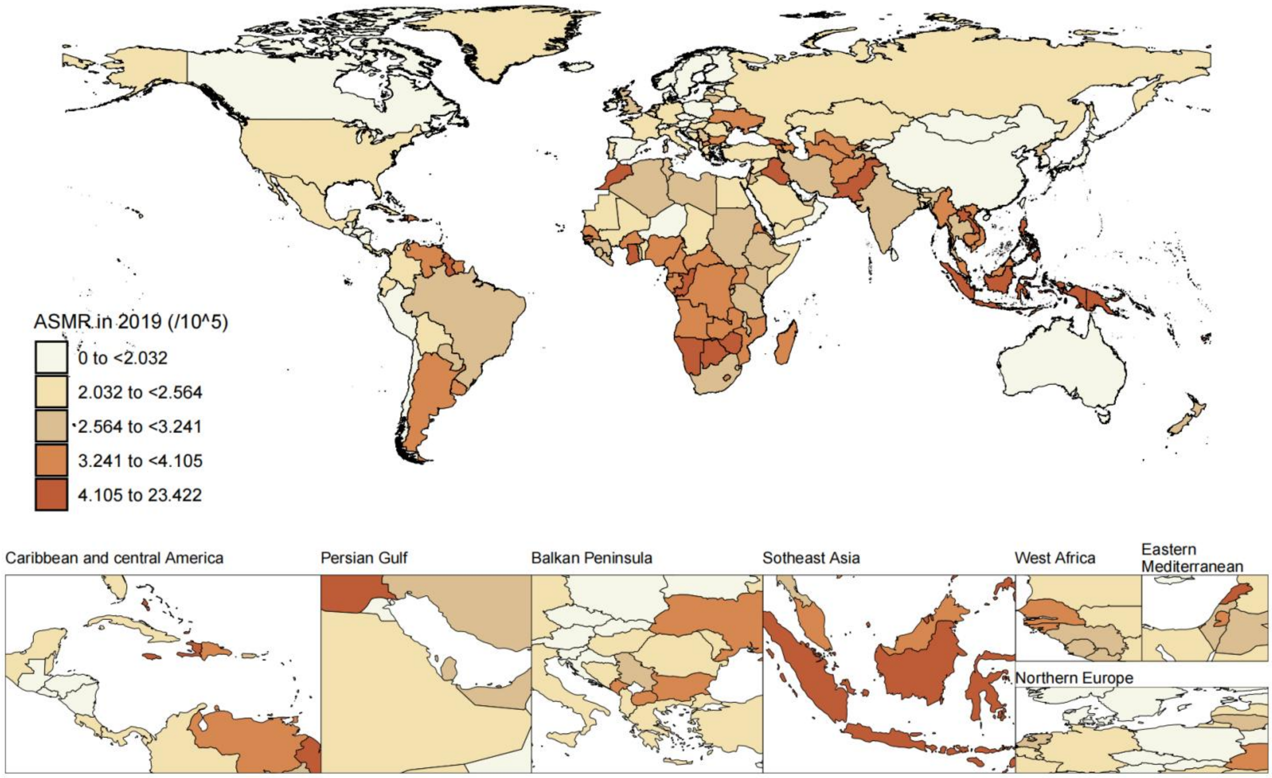

Figure S4: Age-standardised mortality rates of breast cancer in young women in 204 countries and territories, in 2019.

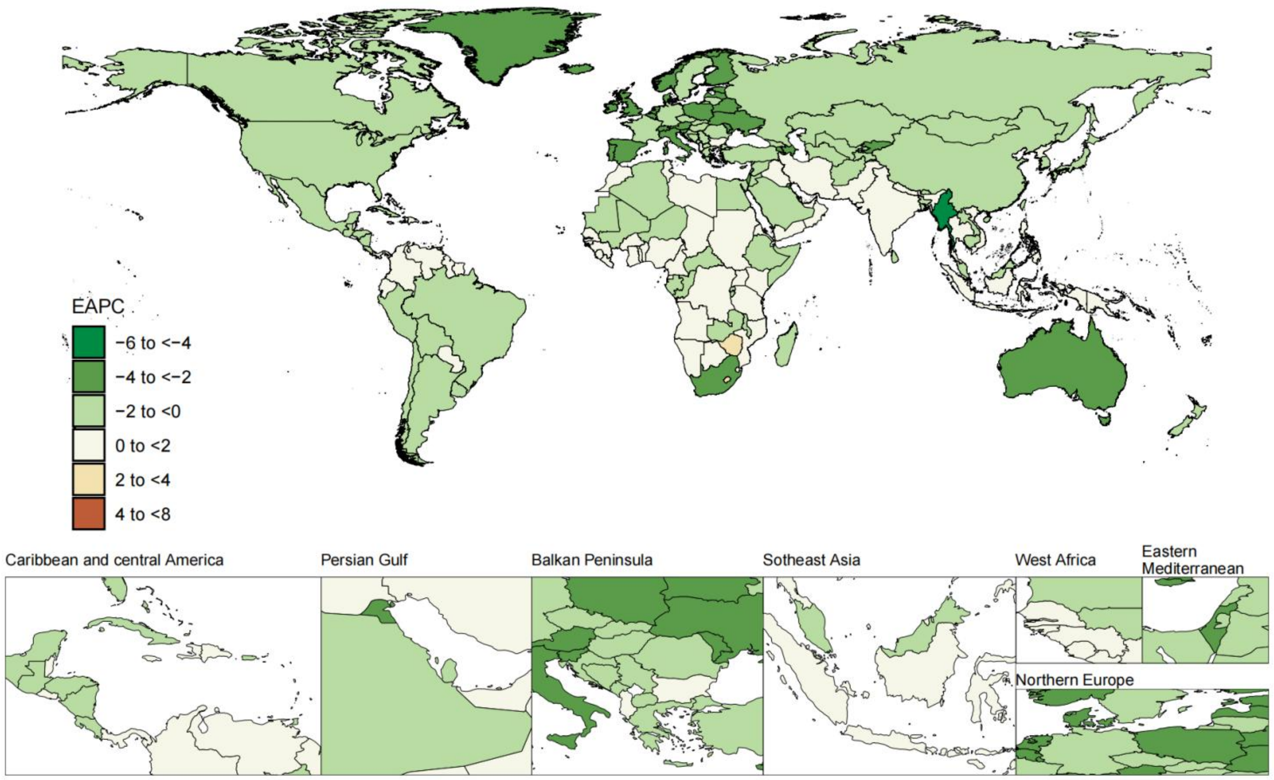

Figure S5: Estimated annual percentage change of age-standardised mortality rates of breast cancer in young women in 204 countries and territories, between 1990 and 2019.

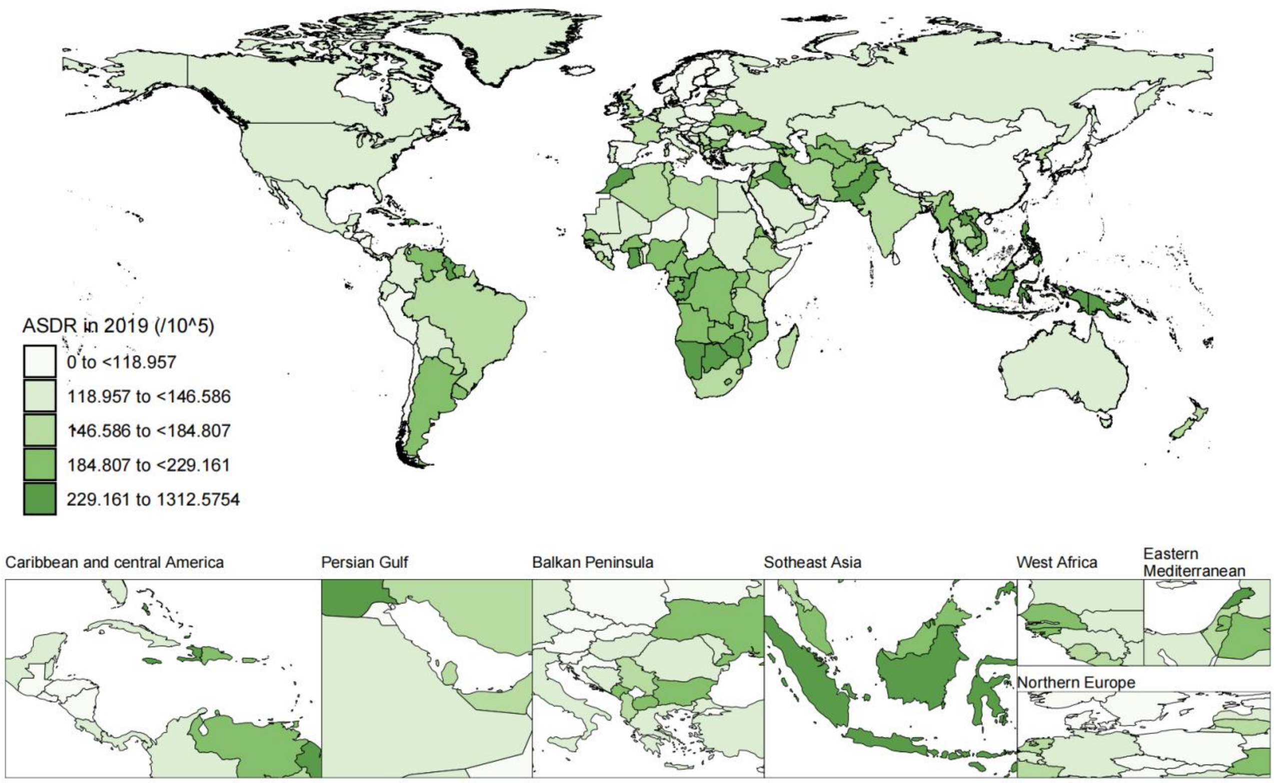

Figure S6: Age-standardised dalys rates of breast cancer in young women in 204 countries and territories, in 2019.

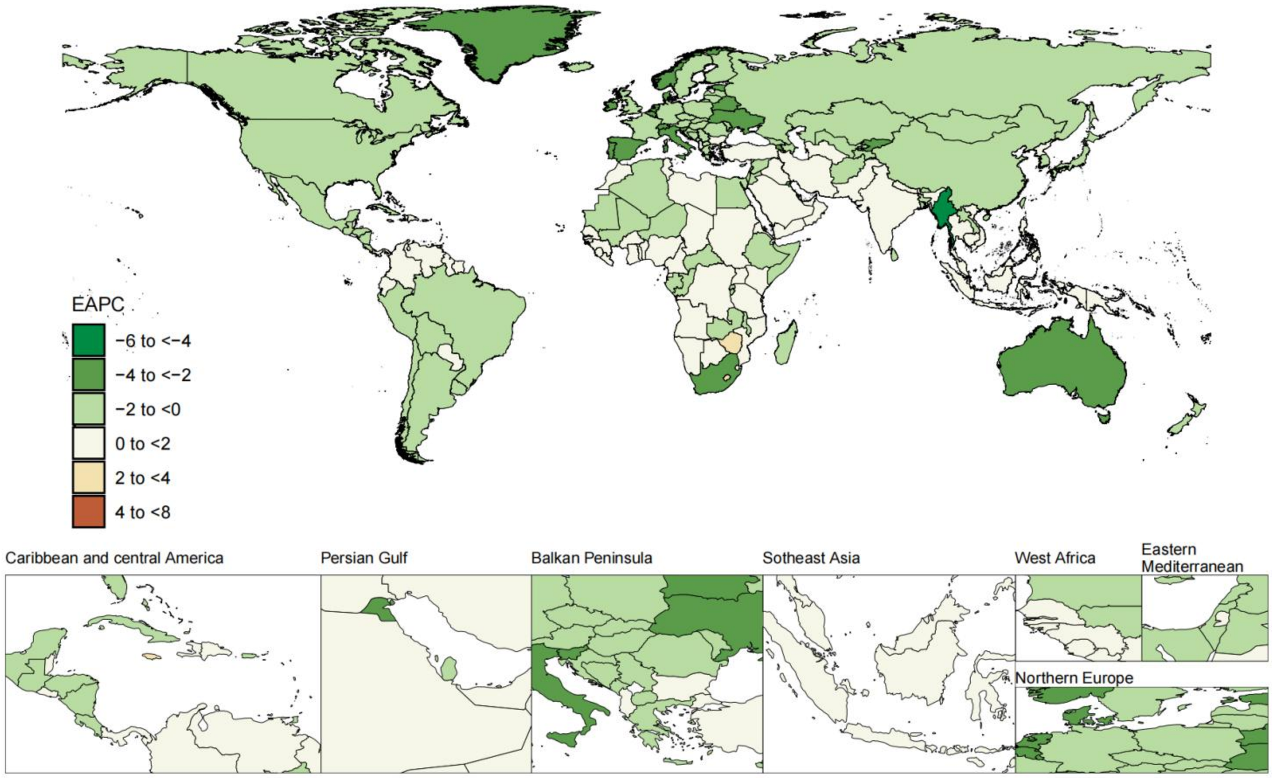

Figure S7: Estimated annual percentage change of age-standardised dalys rates of breast cancer in young women in 204 countries and territories, between 1990 and 2019.

A

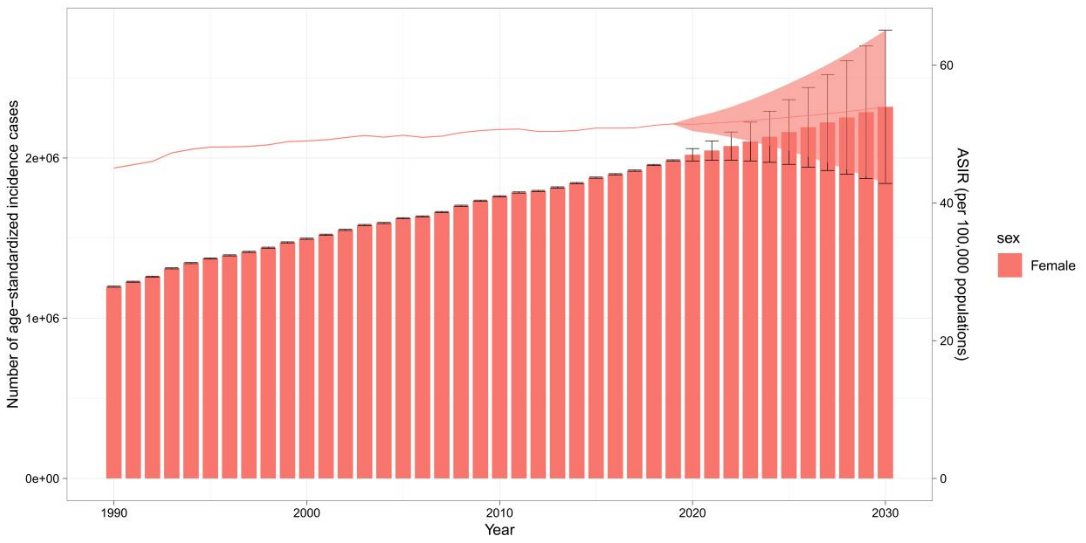

B

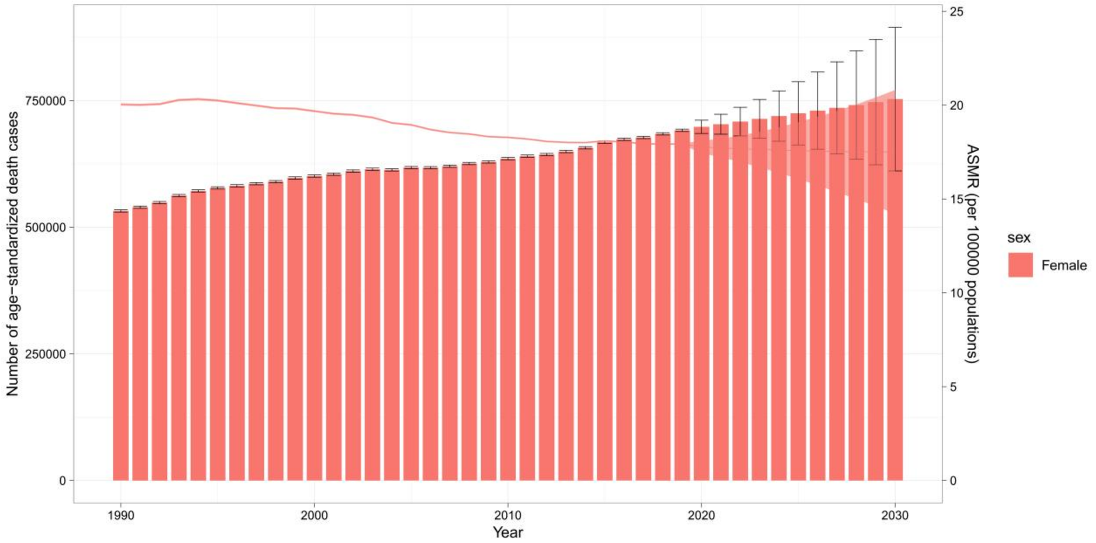

Figure S8: All-age incidence and mortality numbers and rates for female breast cancer patients, 1990-2030. Panel A. All-age incidence. Panel B. All-age mortality.

Note: The pink-shaded area represents the 95% confidence interval of the rate. The black line represents the 95% confidence interval of the number. ASIR – age-standardised incidence rate, ASMR – age-standardised mortality rate.

Table S1: ASIR,ASMR,ASDR of breast cancer in young women among 204 countries in 2019.

location	ASIR per 100,000<br>No. (95% UI)	ASMR per 100,000<br>No. (95% UI)	ASDR per 100,000<br>No. (95% UI)
Afghanistan	8.08(4.82 to 13.02)	4.04(2.41 to 6.46)	228.45(135.97 to 367.26)
Albania	15.04(9.32 to 23.27)	2.55(1.65 to 3.8)	149(96.18 to 222.91)
Algeria	11.89(7.05 to 18.55)	2.67(1.64 to 4.06)	151.82(93.08 to 231.57)
American Samoa	19.08(11.71 to 28.98)	6.14(3.9 to 9.04)	349.14(220.01 to 516.06)
Andorra	20.05(10.81 to 31.18)	2.14(1.22 to 3.18)	130.69(74.13 to 194.95)
Angola	6.35(3.48 to 10.25)	3.29(1.84 to 5.28)	184.88(102.63 to 297.81)
Antigua and Barbuda	13.81(9.64 to 19.35)	3.1(2.25 to 4.25)	177.22(127.99 to 243.02)
Argentina	13.91(9 to 20.3)	3.43(2.66 to 4.28)	197.71(152.49 to 247.49)
Armenia	15.04(10.47 to 20.9)	3.24(2.37 to 4.26)	187.1(137.04 to 245.49)
Australia	17.45(11.46 to 25.55)	1.96(1.55 to 2.45)	119.01(92.97 to 149.29)
Austria	16.63(11.31 to 23.86)	1.83(1.46 to 2.29)	111.38(87.81 to 140.91)
Azerbaijan	11.85(7.44 to 17.92)	3.33(2.17 to 4.91)	189.83(123.05 to 279.93)
Bahamas	31.99(21.14 to 46.7)	8.08(5.6 to 11.25)	459.67(317.77 to 638.83)
Bahrain	17.34(11.06 to 25.64)	3.04(2.05 to 4.33)	175.88(119.39 to 252.79)
Bangladesh	6.29(3.76 to 9.86)	2.34(1.43 to 3.57)	137.06(83.51 to 210.27)
Barbados	24.75(16.9 to 34.82)	5.34(3.92 to 7.17)	305.29(221.4 to 410.58)
Belarus	11.4(6.74 to 17.89)	1.92(1.2 to 2.91)	111.76(69.73 to 168.37)
Belgium	19.72(12.79 to 29.54)	2.28(1.79 to 2.87)	138.58(107.05 to 178.39)
Belize	7.85(5.51 to 10.84)	2.3(1.66 to 3.12)	131.6(94.79 to 178.43)
Benin	4.61(2.51 to 7.94)	2.4(1.32 to 4.1)	134.13(73.14 to 228.69)
Bermuda	19.1(12.37 to 27.78)	2.48(1.69 to 3.45)	147.45(100.38 to 207.25)
Bhutan	5.1(2.38 to 8.93)	1.88(0.92 to 3.26)	105.93(51.62 to 184.85)
Bolivia (Plurinational State of)	6.38(3.32 to 10.22)	2.38(1.24 to 3.83)	133.26(69.58 to 213.74)
Bosnia and Herzegovina	13(8.55 to 19)	2.46(1.67 to 3.45)	141.85(95.9 to 198.71)
Botswana	12.1(6.25 to 20.58)	4.38(2.3 to 7.36)	246.17(129.78 to 414.28)
Brazil	11.25(10.08 to 12.65)	2.85(2.59 to 3.15)	162.46(147.42 to 179.61)
Brunei Darussalam	24.51(15.47 to 36.86)	5.04(3.31 to 7.24)	292.7(191.51 to 420.13)

location	ASIR per 100,000<br>No. (95% UI)	ASMR per 100,000<br>No. (95% UI)	ASDR per 100,000<br>No. (95% UI)
Bulgaria	19.66(12.45 to 29.39)	3.46(2.29 to 5.04)	201.37(132.07 to 290.88)
Burkina Faso	6.97(4.08 to 10.97)	3.6(2.17 to 5.61)	203.28(121.37 to 317.85)
Burundi	5.03(2.78 to 8.37)	2.81(1.54 to 4.6)	156.6(85.8 to 256.75)
Cabo Verde	6.21(3.76 to 9.5)	1.98(1.24 to 2.97)	111.78(69.95 to 167.57)
Cambodia	8.95(5.33 to 14.16)	3.48(2.12 to 5.39)	195.69(119.28 to 302.74)
Cameroon	7.15(3.95 to 11.9)	3.49(1.96 to 5.85)	194.57(109.3 to 327.24)
Canada	18.53(11.71 to 28.23)	2.01(1.55 to 2.56)	123.01(93.44 to 157.71)
Central African Republic	5.22(2.45 to 10.03)	3.38(1.58 to 6.48)	187.39(87.32 to 360.41)
Chad	3.53(1.95 to 5.69)	2.04(1.15 to 3.3)	113.35(63.48 to 183.17)
Chile	9.37(6.08 to 13.99)	1.76(1.37 to 2.23)	102.11(79.04 to 129.71)
China	11.62(8.73 to 14.9)	1.6(1.21 to 2.04)	95.4(73.68 to 119.64)
Colombia	10.99(6.85 to 16.51)	2.21(1.44 to 3.18)	128.26(83.31 to 184.57)
Comoros	6.73(3.09 to 11.22)	3.45(1.6 to 5.73)	192.47(89.2 to 320.68)
Congo	9.22(4.58 to 16.14)	4.67(2.35 to 8.21)	260.18(130.86 to 458.39)
Cook Islands	30.31(6.7 to 50.92)	6.23(1.41 to 10.04)	360.81(83.16 to 583.19)
Costa Rica	12.1(7.66 to 18.27)	2.18(1.44 to 3.17)	125.92(83.31 to 183.42)
Croatia	15.31(9.54 to 23.22)	1.9(1.27 to 2.72)	112.74(75.22 to 162.37)
Cuba	12.33(8.13 to 17.91)	2.16(1.49 to 2.99)	124.69(85.38 to 172.79)
Cyprus	16.11(10.37 to 24.04)	1.59(1.09 to 2.21)	98.7(67.78 to 139.23)
Czechia	13.76(8.98 to 20.08)	1.68(1.19 to 2.31)	100.63(70.28 to 139.08)
C 么 te d'Ivoire	4.83(2.7 to 7.95)	2.53(1.45 to 4.14)	141.01(80.27 to 230.85)
Democratic People's Republic of Korea	8.83(4.49 to 15.44)	2.71(1.39 to 4.77)	153.16(78.53 to 270.6)
Democratic Republic of the Congo	7.37(4.33 to 11.81)	4.04(2.39 to 6.47)	226.26(133.03 to 362.95)
Denmark	15.99(10.32 to 23.93)	1.88(1.47 to 2.39)	113.56(87.77 to 146.52)
Djibouti	5.62(2.56 to 10.19)	2.71(1.26 to 4.84)	150.98(70.25 to 269.54)
Dominica	19.09(11.36 to 29.24)	5.72(3.57 to 8.59)	325.82(202.83 to 490.92)
Dominican Republic	11.98(6.58 to 19.58)	3.55(2.03 to 5.63)	201.02(114.58 to 319.25)

location	ASIR per 100,000<br>No. (95% UI)	ASMR per 100,000<br>No. (95% UI)	ASDR per 100,000<br>No. (95% UI)
Ecuador	8.45(5.53 to 12.59)	2.28(1.53 to 3.33)	129.5(86.93 to 190.36)
Egypt	9.06(5.28 to 14.53)	2.56(1.54 to 4.04)	145(87.01 to 228.86)
El Salvador	7.7(4.63 to 12)	1.91(1.19 to 2.87)	109.2(67.81 to 164)
Equatorial Guinea	8.54(3.85 to 16.1)	3.63(1.65 to 6.81)	204.51(92.48 to 383.02)
Eritrea	6.01(3.37 to 10.02)	3.41(1.91 to 5.72)	188.47(105.18 to 316.43)
Estonia	14.2(8.76 to 21.53)	1.93(1.25 to 2.85)	114.41(72.82 to 169.01)
Eswatini	6.51(3.02 to 11.71)	3.12(1.45 to 5.54)	174.27(80.7 to 309.58)
Ethiopia	5.31(3.52 to 7.8)	2.66(1.83 to 3.78)	149.11(102.71 to 211.63)
Fiji	19.67(11.73 to 30.71)	7.51(4.58 to 11.3)	424.07(259.47 to 642.63)
Finland	16.08(10.17 to 24.17)	1.64(1.23 to 2.14)	101.22(75.15 to 133.88)
France	21.55(13.62 to 32.26)	2.44(1.92 to 3.05)	148.3(114.76 to 188.02)
Gabon	8.52(4.72 to 14.45)	3.71(2.09 to 6.14)	208.66(117.49 to 346.56)
Gambia	2.92(1.65 to 4.77)	1.49(0.84 to 2.4)	81.58(45.97 to 131.56)
Georgia	20.46(13.72 to 28.96)	5.13(3.64 to 6.94)	295.69(209.4 to 399.77)
Germany	18.67(12.05 to 28.09)	2.14(1.7 to 2.65)	129.68(101.73 to 161.83)
Ghana	11.22(6.56 to 17.63)	5.06(3.02 to 7.69)	282.9(168.24 to 431.29)
Greece	19.9(13.2 to 29.2)	2.38(1.98 to 2.86)	144.21(117.7 to 176.08)
Greenland	9.11(5.59 to 14.01)	2.5(1.59 to 3.68)	141.51(90.72 to 209.12)
Grenada	20.17(13.68 to 28.44)	5.51(3.94 to 7.5)	311.06(221.98 to 424.89)
Guam	14.91(9.45 to 21.67)	3.78(2.51 to 5.36)	216.92(143.69 to 309.05)
Guatemala	4.68(3.13 to 6.74)	1.59(1.11 to 2.25)	89.3(62.1 to 126.25)
Guinea	4.61(2.66 to 7.34)	2.65(1.56 to 4.25)	145.32(85.25 to 232.22)
Guinea-Bissau	7.19(4.04 to 12.34)	4.07(2.3 to 6.96)	225.17(126.82 to 384.85)
Guyana	14.2(8.26 to 22.42)	5.12(3.09 to 7.81)	288.11(173.1 to 441.16)
Haiti	12.12(5.86 to 20.77)	6.06(2.97 to 10.58)	336.96(164.78 to 587.54)
Honduras	4.02(2.12 to 7.01)	1.4(0.75 to 2.38)	77.61(41.63 to 131.59)
Hungary	16.38(10.7 to 23.9)	2.47(1.74 to 3.39)	144.42(101.12 to 199.75)
Iceland	17.49(11.82 to 24.58)	1.93(1.43 to 2.55)	117.99(86.6 to 156.58)

location	ASIR per 100,000<br>No. (95% UI)	ASMR per 100,000<br>No. (95% UI)	ASDR per 100,000<br>No. (95% UI)
India	6.77(5.08 to 8.68)	2.75(2.08 to 3.56)	156.39(118.96 to 201.24)
Indonesia	20.17(15.6 to 27.2)	7.49(5.84 to 9.98)	429.57(336.32 to 567.01)
Iran (Islamic Republic of)	14.05(11.13 to 17.54)	2.61(2.3 to 2.93)	152.19(134.04 to 171.76)
Iraq	17.44(10.56 to 27.93)	4.33(2.72 to 6.73)	246.36(154.86 to 384.54)
Ireland	18.44(11.52 to 27.83)	1.9(1.43 to 2.44)	116.77(86.52 to 150.21)
Israel	20.97(13.4 to 31.39)	2.83(2.3 to 3.43)	167.6(133.87 to 204.28)
Italy	21.06(14.65 to 29.14)	2.06(1.9 to 2.23)	127.15(115.46 to 141.08)
Jamaica	23.51(14.67 to 35.43)	5.87(3.79 to 8.56)	334.7(214.71 to 489.68)
Japan	15.73(11.47 to 20.77)	1.47(1.38 to 1.59)	91.75(84.27 to 101.39)
Jordan	16.55(10.38 to 24.98)	3.22(2.14 to 4.68)	185.89(123.41 to 272.35)
Kazakhstan	10.06(7.15 to 13.83)	2.33(1.74 to 3.06)	133.09(99.4 to 175.37)
Kenya	5.78(3.53 to 8.83)	2.74(1.76 to 4.17)	152.09(97.77 to 231.66)
Kiribati	17.09(9.69 to 27.66)	8.79(5.05 to 14.02)	494.03(282.75 to 789.5)
Kuwait	9.35(6.03 to 14.3)	1.25(0.85 to 1.82)	75(50.56 to 109.65)
Kyrgyzstan	5.92(4 to 8.44)	1.66(1.18 to 2.3)	94.06(66.93 to 130.6)
Lao People's Democratic Republic	9.14(4.91 to 15.73)	4.13(2.24 to 7.02)	230.51(124.92 to 393.36)
Latvia	10.89(6.37 to 17.38)	2.33(1.43 to 3.61)	133.74(82.28 to 207.78)
Lebanon	35.72(22.14 to 54.5)	5.13(3.41 to 7.42)	301.34(197.57 to 440.2)
Lesotho	6.91(3.35 to 12.15)	3.68(1.79 to 6.45)	204.37(99.59 to 359.09)
Liberia	5.52(3.09 to 9.41)	2.83(1.6 to 4.78)	158.15(89 to 266.69)
Libya	12.85(6.97 to 21.45)	3.13(1.79 to 4.99)	177.25(100.36 to 281.97)
Lithuania	14.04(8.95 to 20.7)	2.57(1.72 to 3.64)	148.62(99.81 to 211.1)
Luxembourg	19.39(13.7 to 26.56)	2.19(1.71 to 2.74)	133.38(103.43 to 169.2)
Madagascar	5.96(3.32 to 9.33)	3.31(1.9 to 5.16)	184.2(105.19 to 286.61)
Malawi	5.02(2.79 to 8.33)	2.63(1.48 to 4.27)	147.37(82.84 to 240.27)
Malaysia	15.77(9.78 to 23.6)	3.77(2.46 to 5.44)	213.99(139.49 to 309.28)
Maldives	8.3(5.29 to 12.27)	1.63(1.08 to 2.38)	94.74(62.42 to 139.02)
Mali	4.18(2.33 to 6.76)	2.26(1.27 to 3.66)	123.75(69.89 to 201.81)

location	ASIR per 100,000<br>No. (95% UI)	ASMR per 100,000<br>No. (95% UI)	ASDR per 100,000<br>No. (95% UI)
Malta	21.47(14.5 to 30.58)	2.76(2.06 to 3.6)	165.79(122.35 to 218.97)
Marshall Islands	19.69(9.17 to 34.54)	8.86(4.22 to 15.46)	495.61(235.48 to 866.5)
Mauritania	4.89(2.61 to 8.26)	2.16(1.19 to 3.57)	120.97(66.42 to 200.11)
Mauritius	20.07(13.37 to 28.5)	4.56(3.21 to 6.28)	265.63(186.3 to 364.27)
Mexico	9.66(7.47 to 12.26)	2.27(1.79 to 2.87)	130.09(103.35 to 163.11)
Micronesia (Federated States of)	21.01(3.42 to 43.6)	8.28(1.25 to 17.09)	468.19(73.93 to 965.14)
Monaco	41.99(25.76 to 66.56)	4.43(2.88 to 6.84)	272.01(177.49 to 419.82)
Mongolia	5.36(3.13 to 8.66)	1.84(1.1 to 2.88)	103.64(61.38 to 163.5)
Montenegro	22.08(14.64 to 31.49)	3.42(2.42 to 4.62)	201.1(141.77 to 273.14)
Morocco	16.13(9.47 to 27.14)	4.87(2.99 to 8.05)	272.05(166.17 to 451.49)
Mozambique	7.14(3.82 to 11.82)	3.89(2.09 to 6.37)	218.22(116.93 to 357.19)
Myanmar	8.69(5.22 to 13.53)	3.47(2.08 to 5.31)	196.08(117.76 to 301)
Namibia	9.99(5.07 to 17.47)	4.12(2.1 to 7.05)	229.18(116.35 to 391.14)
Nauru	26.59(12.47 to 46.13)	8.65(4.15 to 14.82)	489.99(234.72 to 840.56)
Nepal	4.04(2.29 to 6.36)	1.77(1.01 to 2.8)	98.25(56.13 to 155.45)
Netherlands	25.24(16.01 to 37.47)	2.58(2.03 to 3.17)	158.17(123.34 to 196.36)
New Zealand	21.75(14.44 to 31.27)	2.76(2.22 to 3.36)	165.83(132.89 to 202.86)
Nicaragua	6.01(3.86 to 8.94)	1.43(0.95 to 2.06)	81.53(53.89 to 117.51)
Niger	2.92(1.49 to 5.28)	1.63(0.86 to 2.92)	90.65(47.58 to 162.27)
Nigeria	7.28(4.42 to 11.68)	3.38(2.16 to 5.42)	187.08(120.14 to 297.02)
Niue	23.41(9.21 to 41.09)	5.59(2.26 to 9.53)	321.24(130.59 to 545.59)
North Macedonia	17.83(11.43 to 26.7)	3.27(2.2 to 4.7)	189.23(126.71 to 272.75)
Northern Mariana Islands	22.12(12.98 to 34.59)	4.72(2.9 to 7.17)	272.82(168.26 to 414.84)
Norway	13.14(9.39 to 18.06)	1.52(1.37 to 1.69)	91.67(81.44 to 103.52)
Oman	9.47(5.92 to 14.56)	1.56(1.04 to 2.27)	91.23(60.73 to 133.4)
Pakistan	21.84(14.25 to 32.48)	9.76(6.44 to 14.41)	568.83(377.38 to 837.62)
Palau	25.34(13.76 to 40.29)	6.06(3.42 to 9.52)	348.79(197.31 to 551.2)
Palestine	16.87(11.66 to 23.73)	4.01(2.92 to 5.32)	229.15(166.54 to 304.32)

location	ASIR per 100,000<br>No. (95% UI)	ASMR per 100,000<br>No. (95% UI)	ASDR per 100,000<br>No. (95% UI)
Panama	11.17(6.8 to 17)	2.35(1.47 to 3.47)	135.87(85.04 to 198.89)
Papua New Guinea	24.41(15.08 to 38)	11.91(7.47 to 18.21)	668.2(417.77 to 1022.66)
Paraguay	10.6(6.26 to 16.64)	2.84(1.75 to 4.33)	161.36(98.57 to 246.94)
Peru	7.12(4.11 to 11.49)	1.75(1.04 to 2.74)	100.72(59.61 to 157.94)
Philippines	12.99(8.95 to 18.4)	4.57(3.26 to 6.25)	257.01(185.68 to 348.39)
Poland	11.94(8.23 to 16.71)	1.92(1.44 to 2.56)	112.24(85.83 to 147.31)
Portugal	20.81(13.28 to 31.68)	2.32(1.8 to 2.92)	140.5(107.26 to 178.59)
Puerto Rico	21.39(13.34 to 32.86)	3.15(2.04 to 4.64)	185.92(121.62 to 271.29)
Qatar	18.92(11.86 to 28.78)	2.69(1.75 to 3.9)	158.61(102.87 to 231.64)
Republic of Korea	14.35(9.33 to 21.05)	1.5(1.12 to 1.98)	92.03(67.53 to 123.48)
Republic of Moldova	9.54(6.73 to 13.24)	2.31(1.72 to 3.06)	132.23(98.16 to 175.51)
Romania	12.87(8.65 to 18.69)	2.36(1.67 to 3.21)	136.76(96.71 to 190.04)
Russian Federation	14.81(11.14 to 19.42)	2.43(1.86 to 3.15)	141.67(110.3 to 181.8)
Rwanda	6.72(3.66 to 11.66)	3.3(1.83 to 5.6)	184.52(102.03 to 315.24)
Saint Kitts and Nevis	11.16(1.7 to 22.92)	2.36(0.35 to 4.83)	135.71(21.09 to 274.52)
Saint Lucia	14.5(10.13 to 20.26)	3.72(2.74 to 4.95)	211.56(155.83 to 282.73)
Saint Vincent and the Grenadines	19.19(13.54 to 26.09)	5.58(4.11 to 7.35)	316.77(233.17 to 418.81)
Samoa	11.33(3.91 to 22.05)	3.93(1.44 to 7.36)	218.72(79.23 to 409.94)
San Marino	23.91(14.45 to 36.26)	2.64(1.45 to 4.45)	161.43(90.83 to 266.48)
Sao Tome and Principe	7.27(4 to 12.14)	3.12(1.78 to 5.14)	173.75(98.84 to 287.01)
Saudi Arabia	13.28(7.78 to 21.08)	2.34(1.41 to 3.6)	135.37(82.16 to 207.65)
Senegal	6.79(3.74 to 11.05)	3.39(1.88 to 5.51)	190.52(104.53 to 310.55)
Serbia	17.17(10.95 to 25.76)	2.96(2 to 4.33)	172.9(116.31 to 252.63)
Seychelles	19.98(12.94 to 29.39)	5.16(3.47 to 7.32)	295.62(197.83 to 420.27)
Sierra Leone	5.56(3.12 to 9.24)	2.91(1.65 to 4.84)	161.98(91.06 to 271.1)
Singapore	10.66(6.92 to 15.8)	1.13(0.86 to 1.45)	69.59(52.38 to 90.72)
Slovakia	12.14(7.4 to 18.79)	1.84(1.17 to 2.74)	108.04(68.44 to 160.9)
Slovenia	14.21(8.89 to 21.84)	1.65(1.1 to 2.48)	98.68(65.53 to 148.09)

location	ASIR per 100,000<br>No. (95% UI)	ASMR per 100,000<br>No. (95% UI)	ASDR per 100,000<br>No. (95% UI)
Solomon Islands	55.19(31.94 to 85.34)	23.42(13.88 to 35.64)	1312.58(772.81 to 2004.5)
Somalia	3.07(1.38 to 5.41)	2.12(1.15 to 3.63)	116.7(63.68 to 200.12)
South Africa	7.78(3.92 to 12.35)	3(1.51 to 4.77)	169.73(85.38 to 270.72)
South Sudan	3.4(1.71 to 5.95)	2.05(1.01 to 3.67)	114.14(55.99 to 204.76)
Spain	17.99(11.71 to 26.95)	1.95(1.56 to 2.4)	118.88(93.5 to 148.44)
Sri Lanka	8.69(5.15 to 13.91)	1.8(1.11 to 2.78)	104.36(64.24 to 161.99)
Sudan	7.95(3.75 to 13.49)	2.61(1.27 to 4.48)	146.39(71.09 to 251.88)
Suriname	12.33(7.57 to 19.13)	4.02(2.56 to 6.07)	227.3(144.07 to 343.54)
Sweden	15.66(10.88 to 22.11)	1.76(1.44 to 2.13)	107.37(86.92 to 131.02)
Switzerland	14.23(9.04 to 21.76)	1.51(1.15 to 1.92)	92.38(69.92 to 119.36)
Syrian Arab Republic	9.63(5.79 to 15.08)	2.13(1.33 to 3.24)	123.33(77.36 to 187.64)
Taiwan (Province of China)	16.05(10.35 to 24.36)	1.98(1.35 to 2.85)	119.03(81.1 to 171.86)
Tajikistan	8.65(5.52 to 12.98)	3.21(2.09 to 4.7)	180.49(117.08 to 265.01)
Thailand	13.73(7.66 to 21.62)	2.88(1.67 to 4.4)	167.17(96.63 to 254.4)
Timor-Leste	6.08(0.78 to 10.79)	2.6(0.33 to 4.49)	145.46(18.65 to 251.11)
Togo	5.8(3.37 to 9.38)	2.86(1.68 to 4.51)	159.82(93.82 to 252.17)
Tokelau	22.25(10.66 to 38.89)	6.9(3.37 to 11.82)	390.88(192.7 to 668.5)
Tonga	17.22(9.77 to 27.75)	6.05(3.53 to 9.35)	341.17(198.92 to 528.59)
Trinidad and Tobago	14.54(8.6 to 22.78)	3.88(2.38 to 5.95)	220.82(136 to 339.04)
Tunisia	16.06(8.9 to 25.88)	2.94(1.72 to 4.55)	169.39(99.02 to 261.31)
Turkey	12.88(8.37 to 18.76)	2.27(1.57 to 3.17)	132.64(91.36 to 185.56)
Turkmenistan	11.48(7.79 to 16.6)	3.3(2.35 to 4.6)	188.38(133.61 to 262.29)
Tuvalu	19.53(9.96 to 34.43)	7.33(3.83 to 12.66)	412.64(215.63 to 711.6)
Uganda	8.11(4.67 to 12.8)	4.1(2.37 to 6.54)	227.18(131.51 to 364.34)
Ukraine	14.67(9.37 to 21.39)	3.5(2.33 to 5.07)	200.95(135.07 to 287.9)
United Arab Emirates	11.57(6.17 to 19.36)	2.98(1.65 to 4.81)	167.62(93.08 to 271.12)
United Kingdom	22.45(16.61 to 29.75)	2.75(2.6 to 2.92)	166.4(155.02 to 180.01)
United Republic of Tanzania	5.47(3.14 to 8.87)	2.66(1.58 to 4.2)	150.72(89.11 to 238.94)

location	ASIR per 100,000<br>No. (95% UI)	ASMR per 100,000<br>No. (95% UI)	ASDR per 100,000<br>No. (95% UI)
United States of America	17.98(14 to 23.03)	2.22(2.05 to 2.39)	133.84(122.45 to 145.89)
United States Virgin Islands	13.17(7.49 to 22.2)	3.08(1.85 to 5.05)	175.09(104.56 to 287.09)
Uruguay	15.46(9.72 to 23.33)	3.52(2.65 to 4.54)	202.35(151.24 to 262.67)
Uzbekistan	12.86(9.27 to 17.63)	3.64(2.68 to 4.79)	207.38(152.69 to 273.9)
Vanuatu	12.23(5.25 to 22.26)	5.85(2.55 to 10.43)	326.62(140.61 to 583.01)
Venezuela (Bolivarian Republic of)	15.32(9.69 to 23.11)	3.45(2.27 to 5.02)	199.91(130.82 to 293.23)
Viet Nam	14.66(9.46 to 21.45)	3.72(2.48 to 5.25)	210.73(139.73 to 299.82)
Yemen	6.09(3.04 to 10.44)	2.43(1.25 to 4.09)	135(69.68 to 227.34)
Zambia	7.89(4.46 to 12.33)	3.8(2.19 to 6)	213.68(123.46 to 338.71)
Zimbabwe	9.04(5.25 to 14.71)	4.56(2.65 to 7.26)	252.64(146.33 to 402.81)

ASIR – age standardised incidence rate, ASMR – age standardised mortality rate, ASDR – age standardised DALYs rate, UI – uncertainty interval.

Table S2: Global Burden of Disease in Young Women with Breast Cancer by Age Group 15-39 Years.

Age Group	Incidence No. (95% UI)			Deaths No. (95% UI)			DALYs No. (95% UI)		
	1990	2019	1990-2019	1990	2019	1990-2019	1990	2019	1990-2019
	number		change(%)	number		change(%)	number		change(%)
15-19	1341.25 (1194.46 to 1506.62)	2746.35 (2358.17 to 3247.42)	104.76 (69.06 to 151.77)	409.03 (356.51 to 470.29)	678.07 (556.18 to 815.25)	65.77 (31.33 to 113.58)	30130.8 (26213.73 to 34650.62)	50281.91 (41405.82 to 60734.45)	66.88 (32.23 to 114.63)
20-24	3001.5 (2698.09 to 3282.23)	6380.35 (5598.7 to 7231)	112.57 (78.5 to 152.51)	971.81 (849.97 to 1084.06)	1647.9 (1403.46 to 1897.97)	69.57 (39.66 to 108.18)	66855.4 (58492.36 to 74602.34)	114232.91 (97793.53 to 131093.37)	70.87 (41.38 to 108.9)
25-29	9600.38 (8862.52 to 10290.99)	19263.29 (17144.68 to 21493.46)	100.65 (75.05 to 131.55)	2913.86 (2612.28 to 3193.49)	4492.54 (3965 to 5041.85)	54.18 (31.24 to 82.69)	186622.73 (166664.5 to 204247.08)	290793.22 (256801.92 to 324883.64)	55.82 (33.42 to 83.89)
30-34	25432.61 (23837.01 to 26901.01)	49648 (44746.35 to 54737.58)	95.21 (73.53 to 118.86)	8338.51 (7606.65 to 9050.27)	12057.63 (10886.99 to 13237.52)	44.6 (27.69 to 64.04)	490230.4 (448036.15 to 531459.58)	718167.92 (646797.67 to 786898.79)	46.5 (29.5 to 66.22)
35-39	49798.4 (47070.86 to 52725.11)	90737.86 (82253.81 to 99648.91)	82.21 (62.84 to 101.55)	17126.13 (15966.16 to 18356.83)	23866.22 (21728.92 to 26317.53)	39.36 (24.57 to 54.9)	918709.47 (857429.43 to 984908.45)	1295047.44 (1177289.25 to 1419097.51)	40.96 (26.53 to 56.1)
15-39	89174.14 (83900.84 to 94311.19)	168775.84 (153043.02 to 185086.12)	89.27 (68.57 to 109.26)	29759.35 (27501.2 to 31960.11)	42742.36 (38756.19 to 46959.81)	43.63 (28.7 to 61.72)	1692548.8 (1561128.69 to 1820720.32)	2468523.41 (2238022.2 to 2701471.24)	45.85 (30.83 to 64.1)
	rate/100,000		change(%)	rate/100,000		change(%)	rate/100,000		change(%)
15-19	0.52 (0.47 to 0.59)	0.91 (0.78 to 1.08)	73.39 (43.16 to 113.2)	0.16 (0.14 to 0.18)	0.22 (0.18 to 0.27)	40.38 (11.21 to 80.86)	11.79 (10.26 to 13.56)	16.66 (13.72 to 20.13)	41.31 (11.97 to 81.75)

	rate/100,000		change(%)	rate/100,000		change(%)	rate/100,000		change(%)
20-24	1.23 (1.1 to 1.34)	2.16 (1.89 to 2.44)	75.62 (47.48 to 108.62)	0.4 (0.35 to 0.44)	0.56 (0.47 to 0.64)	40.1 (15.39 to 72)	27.36 (23.94 to 30.53)	38.62 (33.06 to 44.32)	41.17 (16.8 to 72.59)
25-29	4.36 (4.03 to 4.68)	6.41 (5.7 to 7.15)	46.88 (28.14 to 69.5)	1.32 (1.19 to 1.45)	1.49 (1.32 to 1.68)	12.86 (-3.93 to 33.73)	84.79 (75.72 to 92.79)	96.71 (85.4 to 108.04)	14.06 (-2.34 to 34.61)
30-34	13.37 (12.53 to 14.15)	16.63 (14.99 to 18.33)	24.34 (10.53 to 39.4)	4.38 (4 to 4.76)	4.04 (3.65 to 4.43)	-7.9 (-18.67 to 4.49)	257.79 (235.61 to 279.47)	240.55 (216.64 to 263.57)	-6.69 (-17.52 to 5.87)
35-39	28.66 (27.09 to 30.35)	33.8 (30.64 to 37.12)	17.93 (5.39 to 30.44)	9.86 (9.19 to 10.57)	8.89 (8.09 to 9.8)	-9.81 (-19.38 to 0.26)	528.79 (493.52 to 566.89)	482.44 (438.57 to 528.65)	-8.77 (-18.11 to 1.03)
15-39	8.23 (7.74 to 8.7)	11.52 (10.45 to 12.63)	40.01 (24.7 to 54.8)	2.75 (2.54 to 2.95)	2.92 (2.65 to 3.2)	6.25 (-4.8 to 19.63)	156.15 (144.03 to 167.98)	168.47 (152.74 to 184.37)	7.89 (-3.22 to 21.39)

DALYs – disability-adjusted life-years, UI – uncertainty interval.

Table S3: Estimated annual percentage change of ASIR, ASMR, and ASDR for breast cancer in young women in 204 countries.

location	ASIR per 100,000<br>No. (95% CI)	ASMR per 100,000<br>No. (95% CI)	ASDR per 100,000<br>No. (95% CI)
Albania	2.64(2.32 to 2.96)	0.16(-0.12 to 0.45)	0.3(0.02 to 0.59)
Algeria	0.92(0.83 to 1.01)	-0.88(-0.97 to -0.79)	-0.81(-0.9 to -0.73)
American Samoa	2.33(2.16 to 2.51)	1.55(1.41 to 1.68)	1.58(1.44 to 1.72)
Andorra	0.47(0.26 to 0.68)	-1.13(-1.21 to -1.05)	-0.97(-1.05 to -0.9)
Angola	1.47(1.33 to 1.6)	0.57(0.45 to 0.69)	0.6(0.48 to 0.73)
Antigua and Barbuda	0.23(-0.08 to 0.54)	-0.85(-1.14 to -0.56)	-0.81(-1.11 to -0.52)
Argentina	0.36(0.17 to 0.55)	-1.08(-1.28 to -0.87)	-1(-1.2 to -0.79)
Armenia	-1.71(-2.13 to -1.28)	-3.44(-3.9 to -2.98)	-3.36(-3.81 to -2.91)
Australia	-0.35(-0.5 to -0.2)	-2.21(-2.41 to -2)	-2.02(-2.21 to -1.84)
Austria	-0.36(-0.58 to -0.14)	-2(-2.27 to -1.72)	-1.83(-2.08 to -1.57)
Azerbaijan	-0.6(-0.95 to -0.25)	-2.04(-2.29 to -1.79)	-1.98(-2.24 to -1.73)
Bahamas	0.43(0.26 to 0.61)	-0.58(-0.83 to -0.33)	-0.54(-0.78 to -0.3)
Bahrain	1.98(1.26 to 2.71)	-0.73(-1.44 to -0.02)	-0.57(-1.27 to 0.13)
Bangladesh	-0.2(-0.41 to 0.01)	-1.78(-2.02 to -1.54)	-1.72(-1.95 to -1.48)
Barbados	0.76(0.5 to 1.02)	-0.31(-0.55 to -0.07)	-0.26(-0.5 to -0.02)
Belarus	-1.5(-1.75 to -1.24)	-3.48(-3.72 to -3.24)	-3.37(-3.61 to -3.14)
Belgium	-0.42(-0.59 to -0.25)	-2.27(-2.36 to -2.17)	-2.09(-2.18 to -2)
Belize	1.91(1.38 to 2.44)	0.86(0.31 to 1.42)	0.95(0.41 to 1.5)
Benin	1.25(1.14 to 1.36)	0.65(0.53 to 0.77)	0.67(0.55 to 0.79)
Bermuda	0.05(-0.12 to 0.22)	-2.15(-2.42 to -1.88)	-1.99(-2.26 to -1.73)
Bhutan	0.6(0.38 to 0.82)	-1.34(-1.57 to -1.12)	-1.29(-1.52 to -1.07)
Bolivia (Plurinational State of)	0.49(0.34 to 0.65)	-0.98(-1.12 to -0.85)	-0.95(-1.08 to -0.82)
Bosnia and Herzegovina	1.85(1.66 to 2.05)	-0.49(-0.68 to -0.3)	-0.36(-0.53 to -0.18)
Botswana	2.98(2.72 to 3.24)	1.73(1.5 to 1.96)	1.77(1.54 to 1.99)
Brazil	1.26(1.15 to 1.38)	-0.23(-0.31 to -0.15)	-0.17(-0.25 to -0.09)
Brunei Darussalam	1.79(1.49 to 2.09)	0.26(-0.1 to 0.62)	0.37(0.01 to 0.73)
Bulgaria	0.99(0.52 to 1.45)	0.08(-0.29 to 0.44)	0.14(-0.23 to 0.51)

location	ASIR per 100,000<br>No. (95% CI)	ASMR per 100,000<br>No. (95% CI)	ASDR per 100,000<br>No. (95% CI)
Burkina Faso	0.96(0.81 to 1.11)	0.35(0.15 to 0.54)	0.37(0.18 to 0.56)
Burundi	-0.37(-0.51 to -0.23)	-1.05(-1.23 to -0.87)	-1.02(-1.2 to -0.85)
Cabo Verde	0.23(-0.1 to 0.56)	-1.46(-1.72 to -1.2)	-1.43(-1.69 to -1.16)
Cambodia	1.47(1.4 to 1.55)	-0.02(-0.13 to 0.09)	0.01(-0.1 to 0.12)
Cameroon	1.39(1.3 to 1.48)	0.65(0.54 to 0.75)	0.67(0.56 to 0.78)
Canada	-0.21(-0.32 to -0.1)	-1.68(-1.83 to -1.52)	-1.5(-1.66 to -1.35)
Central African Republic	-0.01(-0.1 to 0.09)	-0.18(-0.28 to -0.08)	-0.18(-0.28 to -0.09)
Chad	0.85(0.8 to 0.9)	0.48(0.4 to 0.56)	0.5(0.42 to 0.58)
Chile	1.68(1.45 to 1.92)	-0.72(-1.01 to -0.43)	-0.58(-0.87 to -0.3)
China	2.29(2 to 2.57)	-1.44(-1.8 to -1.07)	-1.22(-1.59 to -0.85)
Colombia	2.1(1.83 to 2.37)	0.11(-0.23 to 0.46)	0.22(-0.12 to 0.57)
Comoros	1.52(0.95 to 2.1)	0.62(0.04 to 1.21)	0.65(0.05 to 1.25)
Congo	0.77(0.58 to 0.96)	-0.05(-0.26 to 0.16)	-0.04(-0.24 to 0.17)
Cook Islands	0.27(-0.02 to 0.56)	-0.93(-1.25 to -0.61)	-0.87(-1.18 to -0.56)
Costa Rica	1.12(0.78 to 1.46)	-0.24(-0.61 to 0.12)	-0.15(-0.5 to 0.21)
Croatia	-0.12(-0.5 to 0.27)	-1.82(-2.18 to -1.45)	-1.68(-2.04 to -1.31)
Cuba	-0.15(-0.39 to 0.1)	-1.46(-1.75 to -1.17)	-1.39(-1.68 to -1.11)
Cyprus	1.1(0.37 to 1.82)	-2.11(-2.58 to -1.64)	-1.79(-2.27 to -1.31)
Czechia	0.1(-0.17 to 0.37)	-1.72(-2.11 to -1.32)	-1.56(-1.94 to -1.18)
C 么 te d'Ivoire	1.41(1.28 to 1.54)	0.91(0.79 to 1.03)	0.92(0.8 to 1.04)
Democratic People's Republic of Korea	0.6(0.44 to 0.77)	0.18(0.12 to 0.25)	0.2(0.15 to 0.26)
Democratic Republic of the Congo	0.62(0.39 to 0.85)	0.18(-0.01 to 0.37)	0.18(-0.01 to 0.37)
Denmark	-0.87(-1.05 to -0.68)	-3.27(-3.46 to -3.08)	-3.04(-3.23 to -2.86)
Djibouti	1.68(1.53 to 1.84)	0.76(0.63 to 0.89)	0.79(0.66 to 0.92)
Dominica	-0.26(-0.67 to 0.16)	-0.82(-1.21 to -0.43)	-0.75(-1.12 to -0.38)
Dominican Republic	2.53(2.07 to 2.98)	1.33(0.98 to 1.69)	1.36(1.01 to 1.71)
Ecuador	2.49(2.29 to 2.7)	0.51(0.32 to 0.7)	0.58(0.39 to 0.77)

location	ASIR per 100,000<br>No. (95% CI)	ASMR per 100,000<br>No. (95% CI)	ASDR per 100,000<br>No. (95% CI)
Egypt	1.35(1.16 to 1.54)	-0.24(-0.36 to -0.12)	-0.18(-0.3 to -0.06)
El Salvador	2.55(2.23 to 2.87)	0.6(0.41 to 0.8)	0.69(0.49 to 0.89)
Equatorial Guinea	2.96(2.72 to 3.2)	1.09(0.91 to 1.27)	1.15(0.98 to 1.32)
Eritrea	1.81(1.7 to 1.91)	1.12(1.04 to 1.21)	1.14(1.05 to 1.23)
Estonia	0.3(-0.12 to 0.71)	-2.55(-3.02 to -2.07)	-2.33(-2.8 to -1.86)
Eswatini	1.86(1.58 to 2.13)	1.4(1.04 to 1.77)	1.42(1.06 to 1.78)
Ethiopia	-0.58(-0.87 to -0.29)	-1.58(-1.83 to -1.33)	-1.56(-1.81 to -1.32)
Fiji	0.97(0.7 to 1.24)	0.45(0.21 to 0.69)	0.46(0.22 to 0.71)
Finland	0.08(-0.07 to 0.24)	-2.16(-2.3 to -2.02)	-1.9(-2.03 to -1.77)
France	0.8(0.5 to 1.11)	-1.38(-1.55 to -1.21)	-1.18(-1.35 to -1.01)
Gabon	0.59(0.44 to 0.74)	-0.32(-0.53 to -0.11)	-0.29(-0.5 to -0.09)
Gambia	1.84(1.65 to 2.02)	1.34(1.17 to 1.52)	1.33(1.16 to 1.51)
Georgia	-1(-1.37 to -0.62)	-1.26(-1.65 to -0.88)	-1.25(-1.62 to -0.87)
Germany	0.22(0.11 to 0.34)	-1.97(-2.27 to -1.68)	-1.77(-2.05 to -1.48)
Ghana	0.77(0.63 to 0.9)	0.05(-0.06 to 0.16)	0.07(-0.05 to 0.18)
Greece	0.14(-0.06 to 0.35)	-1.23(-1.45 to -1.02)	-1.12(-1.33 to -0.92)
Greenland	-1.08(-1.32 to -0.85)	-2.23(-2.43 to -2.02)	-2.17(-2.38 to -1.97)
Grenada	0.16(-0.17 to 0.5)	-0.8(-1.19 to -0.4)	-0.76(-1.14 to -0.38)
Guam	1.68(1.3 to 2.07)	1.42(1.21 to 1.62)	1.4(1.19 to 1.6)
Guatemala	1.19(0.93 to 1.44)	-0.39(-0.7 to -0.08)	-0.34(-0.64 to -0.03)
Guinea	0.77(0.66 to 0.88)	0.34(0.2 to 0.48)	0.33(0.19 to 0.47)
Guinea-Bissau	1.2(1.12 to 1.28)	0.77(0.69 to 0.85)	0.77(0.69 to 0.85)
Guyana	0.9(0.42 to 1.39)	0.38(-0.07 to 0.83)	0.38(-0.07 to 0.83)
Haiti	0.77(0.64 to 0.9)	0.12(0.01 to 0.24)	0.13(0.01 to 0.25)
Honduras	-0.59(-0.92 to -0.26)	-1.71(-2.03 to -1.4)	-1.72(-2.03 to -1.41)
Hungary	-0.21(-0.47 to 0.04)	-1.96(-2.32 to -1.6)	-1.82(-2.17 to -1.48)
Iceland	-0.81(-0.99 to -0.64)	-2.13(-2.35 to -1.92)	-1.98(-2.18 to -1.79)
India	1.65(1.51 to 1.78)	0.45(0.32 to 0.59)	0.48(0.35 to 0.62)

location	ASIR per 100,000<br>No. (95% CI)	ASMR per 100,000<br>No. (95% CI)	ASDR per 100,000<br>No. (95% CI)
Indonesia	1.27(1.15 to 1.39)	0.18(0.03 to 0.33)	0.22(0.07 to 0.37)
Iran (Islamic Republic of)	2.48(2.29 to 2.67)	0.73(0.53 to 0.93)	0.83(0.64 to 1.02)
Iraq	2.25(2.15 to 2.35)	0.28(0.04 to 0.52)	0.37(0.14 to 0.6)
Ireland	0.05(-0.22 to 0.33)	-2.51(-2.71 to -2.3)	-2.24(-2.44 to -2.05)
Israel	0.04(-0.19 to 0.27)	-2.04(-2.31 to -1.77)	-1.91(-2.17 to -1.64)
Italy	-0.77(-0.9 to -0.64)	-2.51(-2.69 to -2.34)	-2.34(-2.5 to -2.18)
Jamaica	3.27(2.75 to 3.79)	2(1.48 to 2.51)	2.07(1.56 to 2.59)
Japan	0.51(0.29 to 0.72)	-1.35(-1.48 to -1.21)	-1.14(-1.27 to -1)
Jordan	1.69(1.31 to 2.07)	-0.86(-1.34 to -0.37)	-0.72(-1.19 to -0.24)
Kazakhstan	-0.05(-0.26 to 0.17)	-1.82(-2.02 to -1.63)	-1.76(-1.95 to -1.58)
Kenya	1.56(1.42 to 1.7)	1.51(1.43 to 1.59)	1.51(1.43 to 1.59)
Kiribati	0.34(0.27 to 0.41)	-0.05(-0.11 to 0)	-0.04(-0.1 to 0.01)
Kuwait	-1.37(-1.91 to -0.83)	-3.27(-3.84 to -2.7)	-3.13(-3.69 to -2.58)
Kyrgyzstan	-2.31(-2.7 to -1.91)	-3.57(-3.89 to -3.25)	-3.53(-3.85 to -3.21)
Lao People's Democratic Republic	1.04(0.99 to 1.08)	-0.17(-0.28 to -0.07)	-0.12(-0.23 to -0.02)
Latvia	-0.32(-0.68 to 0.05)	-2.07(-2.42 to -1.72)	-1.98(-2.32 to -1.65)
Lebanon	2.91(2.81 to 3.02)	-0.26(-0.37 to -0.15)	-0.06(-0.18 to 0.05)
Lesotho	3.73(3.21 to 4.26)	3.47(2.95 to 4)	3.46(2.94 to 3.98)
Liberia	1.48(1.31 to 1.65)	0.65(0.44 to 0.86)	0.68(0.48 to 0.88)
Libya	2.13(1.68 to 2.58)	0.53(0.13 to 0.92)	0.61(0.22 to 1.01)
Lithuania	0.02(-0.43 to 0.46)	-1.28(-1.77 to -0.8)	-1.19(-1.66 to -0.71)
Luxembourg	-0.52(-0.81 to -0.23)	-2.57(-2.77 to -2.37)	-2.38(-2.57 to -2.18)
Madagascar	-0.04(-0.32 to 0.25)	-0.43(-0.72 to -0.14)	-0.43(-0.72 to -0.14)
Malawi	0.37(0.25 to 0.5)	-0.32(-0.46 to -0.18)	-0.27(-0.41 to -0.13)
Malaysia	1.97(1.73 to 2.2)	-0.03(-0.32 to 0.26)	0.04(-0.24 to 0.33)
Maldives	-0.03(-0.28 to 0.22)	-3.24(-3.54 to -2.94)	-3.09(-3.38 to -2.8)
Mali	-0.86(-1.18 to -0.54)	-1.47(-1.78 to -1.17)	-1.49(-1.8 to -1.18)
Malta	1.67(1.33 to 2.02)	-0.31(-0.59 to -0.03)	-0.16(-0.44 to 0.12)

location	ASIR per 100,000<br>No. (95% CI)	ASMR per 100,000<br>No. (95% CI)	ASDR per 100,000<br>No. (95% CI)
Marshall Islands	2.13(1.99 to 2.28)	1.58(1.38 to 1.78)	1.6(1.4 to 1.8)
Mauritania	0.95(0.81 to 1.1)	-0.06(-0.15 to 0.04)	-0.03(-0.12 to 0.07)
Mauritius	2.91(2.7 to 3.11)	1.81(1.59 to 2.04)	1.88(1.67 to 2.09)
Mexico	0.9(0.74 to 1.06)	-0.53(-0.69 to -0.38)	-0.47(-0.63 to -0.32)
Micronesia (Federated States of)	1.97(1.75 to 2.19)	0.89(0.68 to 1.1)	0.96(0.76 to 1.16)
Monaco	1.38(1.19 to 1.57)	-0.04(-0.12 to 0.05)	0.1(0.01 to 0.19)
Mongolia	1.36(1.1 to 1.62)	-0.25(-0.6 to 0.1)	-0.21(-0.56 to 0.14)
Montenegro	1.03(0.72 to 1.34)	-0.36(-0.8 to 0.08)	-0.27(-0.69 to 0.16)
Morocco	2.01(1.8 to 2.21)	0.42(0.16 to 0.68)	0.46(0.2 to 0.72)
Mozambique	1.72(1.3 to 2.15)	1.02(0.58 to 1.46)	1.05(0.61 to 1.48)
Myanmar	-3.73(-4.12 to -3.33)	-4.98(-5.4 to -4.56)	-4.95(-5.36 to -4.53)
Namibia	2.85(2.7 to 2.99)	1.56(1.39 to 1.72)	1.58(1.42 to 1.75)
Nauru	1.82(1.68 to 1.96)	1.21(1.12 to 1.31)	1.24(1.16 to 1.33)
Nepal	1.52(1.33 to 1.72)	0.24(0.04 to 0.44)	0.26(0.06 to 0.45)
Netherlands	-0.42(-0.61 to -0.23)	-2.3(-2.52 to -2.08)	-2.1(-2.31 to -1.89)
New Zealand	-0.2(-0.38 to -0.02)	-1.94(-2.08 to -1.79)	-1.81(-1.95 to -1.66)
Nicaragua	1.97(1.55 to 2.4)	-0.1(-0.45 to 0.26)	-0.03(-0.38 to 0.33)
Niger	0.16(0.03 to 0.29)	-0.48(-0.64 to -0.31)	-0.46(-0.63 to -0.29)
Nigeria	1.71(1.56 to 1.85)	0.77(0.61 to 0.92)	0.81(0.65 to 0.96)
Niue	1.53(1.38 to 1.68)	0.04(-0.06 to 0.14)	0.11(0.01 to 0.21)
North Macedonia	0.45(0.21 to 0.69)	-1.57(-1.79 to -1.35)	-1.46(-1.68 to -1.24)
Northern Mariana Islands	-0.04(-0.18 to 0.1)	-0.64(-0.81 to -0.48)	-0.61(-0.77 to -0.45)
Norway	-0.58(-0.9 to -0.25)	-2.89(-3.1 to -2.67)	-2.68(-2.9 to -2.46)
Oman	3.12(2.44 to 3.81)	0.74(0.24 to 1.25)	0.9(0.39 to 1.41)
Pakistan	2.58(2.44 to 2.72)	1.6(1.38 to 1.82)	1.66(1.45 to 1.87)
Palau	1.11(1.03 to 1.19)	0.18(0.13 to 0.23)	0.23(0.18 to 0.28)
Palestine	1.36(0.94 to 1.79)	-0.01(-0.32 to 0.29)	0.05(-0.26 to 0.36)
Panama	1.31(0.98 to 1.64)	0.11(-0.23 to 0.44)	0.19(-0.14 to 0.52)

location	ASIR per 100,000<br>No. (95% CI)	ASMR per 100,000<br>No. (95% CI)	ASDR per 100,000<br>No. (95% CI)
Papua New Guinea	0.69(0.63 to 0.74)	0.34(0.28 to 0.39)	0.34(0.29 to 0.4)
Paraguay	1.34(1.15 to 1.54)	0.06(-0.1 to 0.23)	0.1(-0.06 to 0.26)
Peru	0.46(0.19 to 0.73)	-1.84(-2.12 to -1.55)	-1.73(-2.01 to -1.45)
Philippines	0.99(0.7 to 1.28)	0.31(0.04 to 0.58)	0.35(0.08 to 0.62)
Poland	0.42(0.29 to 0.55)	-2(-2.18 to -1.81)	-1.84(-2.03 to -1.65)
Portugal	-0.41(-0.71 to -0.1)	-2.75(-2.94 to -2.55)	-2.57(-2.77 to -2.37)
Puerto Rico	0.8(0.52 to 1.08)	-1.11(-1.48 to -0.74)	-0.99(-1.35 to -0.63)
Qatar	2.55(2.07 to 3.04)	-0.84(-1.37 to -0.31)	-0.64(-1.16 to -0.11)
Republic of Korea	2.83(2.44 to 3.22)	-0.76(-0.92 to -0.61)	-0.52(-0.68 to -0.37)
Republic of Moldova	-0.65(-0.97 to -0.34)	-2.05(-2.36 to -1.73)	-1.98(-2.29 to -1.67)
Romania	0.4(0.18 to 0.61)	-1.65(-1.92 to -1.37)	-1.52(-1.78 to -1.25)
Russian Federation	1.1(0.76 to 1.45)	-0.94(-1.31 to -0.56)	-0.81(-1.17 to -0.44)
Rwanda	-0.54(-0.89 to -0.18)	-1.75(-2.09 to -1.4)	-1.7(-2.04 to -1.36)
Saint Kitts and Nevis	-3.98(-4.41 to -3.55)	-5.17(-5.68 to -4.67)	-5.11(-5.61 to -4.6)
Saint Lucia	0.53(0.11 to 0.95)	-0.64(-1.11 to -0.17)	-0.58(-1.04 to -0.11)
Saint Vincent and the Grenadines	-0.04(-0.36 to 0.29)	-0.65(-0.97 to -0.33)	-0.66(-0.99 to -0.33)
Samoa	1.14(0.98 to 1.29)	0.47(0.4 to 0.54)	0.49(0.42 to 0.56)
San Marino	2.18(1.97 to 2.39)	0.82(0.63 to 1.01)	0.97(0.78 to 1.16)
Sao Tome and Principe	2.37(1.95 to 2.78)	1.31(0.85 to 1.77)	1.34(0.88 to 1.8)
Saudi Arabia	4.02(3.86 to 4.18)	-0.02(-0.26 to 0.22)	0.16(-0.07 to 0.39)
Senegal	1.04(0.85 to 1.23)	0.39(0.2 to 0.57)	0.41(0.22 to 0.59)
Serbia	0.36(0.13 to 0.59)	-1.79(-2.06 to -1.53)	-1.64(-1.89 to -1.38)
Seychelles	2.72(2.48 to 2.97)	1.17(0.96 to 1.37)	1.25(1.04 to 1.46)
Sierra Leone	2.38(2.22 to 2.54)	1.74(1.61 to 1.87)	1.77(1.63 to 1.9)
Singapore	-0.77(-1.18 to -0.36)	-3.43(-3.75 to -3.11)	-3.16(-3.46 to -2.86)
Slovakia	0.27(0 to 0.55)	-1.81(-2.09 to -1.52)	-1.64(-1.93 to -1.36)
Slovenia	-0.22(-0.41 to -0.03)	-2.33(-2.53 to -2.12)	-2.15(-2.34 to -1.95)
Solomon Islands	8.25(7.41 to 9.09)	7.57(6.69 to 8.45)	7.59(6.72 to 8.47)

location	ASIR per 100,000<br>No. (95% CI)	ASMR per 100,000<br>No. (95% CI)	ASDR per 100,000<br>No. (95% CI)
Somalia	-0.16(-0.3 to -0.01)	-0.19(-0.28 to -0.1)	-0.18(-0.27 to -0.09)
South Africa	-1.65(-2.51 to -0.79)	-2.44(-3.43 to -1.44)	-2.42(-3.42 to -1.41)
South Sudan	0.11(-0.15 to 0.38)	-0.22(-0.49 to 0.04)	-0.21(-0.47 to 0.06)
Spain	-1.1(-1.24 to -0.96)	-3.02(-3.24 to -2.79)	-2.85(-3.07 to -2.64)
Sri Lanka	1.97(1.59 to 2.35)	-0.26(-0.54 to 0.02)	-0.17(-0.45 to 0.12)
Sudan	2.59(2.49 to 2.69)	0.96(0.87 to 1.05)	1.01(0.92 to 1.1)
Suriname	1.28(0.75 to 1.83)	0.43(-0.07 to 0.93)	0.46(-0.04 to 0.96)
Sweden	-0.1(-0.26 to 0.07)	-1.62(-1.78 to -1.46)	-1.44(-1.59 to -1.29)
Switzerland	-1.7(-1.92 to -1.47)	-3.41(-3.73 to -3.09)	-3.26(-3.56 to -2.96)
Syrian Arab Republic	2.11(1.7 to 2.53)	-0.18(-0.67 to 0.32)	-0.04(-0.52 to 0.44)
Taiwan (Province of China)	1.19(0.82 to 1.56)	-1.24(-1.45 to -1.03)	-1.06(-1.27 to -0.85)
Tajikistan	-0.59(-0.95 to -0.24)	-1.23(-1.52 to -0.92)	-1.21(-1.52 to -0.91)
Thailand	2.36(1.35 to 3.38)	0.04(-0.89 to 0.97)	0.12(-0.82 to 1.07)
Timor-Leste	2.03(1.44 to 2.63)	0.68(0.12 to 1.24)	0.72(0.16 to 1.28)
Togo	0.96(0.72 to 1.21)	0.36(0.14 to 0.58)	0.37(0.16 to 0.59)
Tokelau	2.01(1.95 to 2.06)	0.55(0.5 to 0.59)	0.61(0.56 to 0.65)
Tonga	0.69(0.56 to 0.81)	-0.05(-0.19 to 0.09)	-0.01(-0.15 to 0.13)
Trinidad and Tobago	0.24(-0.08 to 0.56)	-0.97(-1.27 to -0.66)	-0.91(-1.21 to -0.6)
Tunisia	2.47(2.27 to 2.68)	0.27(0.1 to 0.43)	0.38(0.22 to 0.54)
Turkey	3.38(3.02 to 3.75)	0(-0.29 to 0.3)	0.17(-0.12 to 0.47)
Turkmenistan	1.41(0.9 to 1.92)	-0.03(-0.51 to 0.46)	0.01(-0.48 to 0.49)
Tuvalu	1.29(1.23 to 1.36)	0.39(0.31 to 0.47)	0.42(0.34 to 0.5)
Uganda	1.32(0.88 to 1.76)	0.54(0.06 to 1.01)	0.55(0.08 to 1.03)
Ukraine	-1.42(-2.01 to -0.83)	-2.85(-3.5 to -2.2)	-2.79(-3.43 to -2.14)
United Arab Emirates	1.57(0.83 to 2.3)	0.3(-0.45 to 1.05)	0.36(-0.38 to 1.11)
United Kingdom	-0.1(-0.29 to 0.1)	-2(-2.25 to -1.75)	-1.82(-2.07 to -1.57)
United Republic of Tanzania	1.51(1.34 to 1.68)	0.89(0.71 to 1.06)	0.88(0.7 to 1.06)
United States of America	-0.97(-1.13 to -0.82)	-1.99(-2.21 to -1.77)	-1.87(-2.09 to -1.66)

location	ASIR per 100,000<br>No. (95% CI)	ASMR per 100,000<br>No. (95% CI)	ASDR per 100,000<br>No. (95% CI)
United States Virgin Islands	0.28(0.1 to 0.46)	-0.92(-1.01 to -0.84)	-0.88(-0.97 to -0.79)
Uruguay	-0.16(-0.32 to 0)	-1.59(-1.73 to -1.46)	-1.51(-1.64 to -1.38)
Uzbekistan	0.8(0.57 to 1.03)	-0.3(-0.57 to -0.03)	-0.27(-0.54 to 0)
Vanuatu	2.05(1.83 to 2.28)	1.72(1.51 to 1.93)	1.73(1.52 to 1.94)
Venezuela (Bolivarian Republic of)	2.33(1.92 to 2.75)	0.63(0.11 to 1.15)	0.75(0.23 to 1.28)
Viet Nam	2.91(2.68 to 3.14)	0.68(0.5 to 0.87)	0.76(0.58 to 0.95)
Yemen	3.19(3 to 3.39)	1.96(1.79 to 2.12)	2(1.84 to 2.17)
Zambia	0.59(0.45 to 0.72)	-0.36(-0.48 to -0.24)	-0.33(-0.45 to -0.21)
Zimbabwe	2.51(2.08 to 2.95)	3.57(2.75 to 4.41)	3.54(2.72 to 4.35)

ASIR – age standardised incidence rate, ASMR – age standardised mortality rate, ASDR – age standardised DALYs rate, CI – confidence intervals.

Table S4: The global and five SDI area disease burden for young breast cancer incidence and mortality rates and changes in 2020-2030.

location	Incidence			Deaths		
	rate/100,000		change(%)	rate/100,000		change(%)
	2020	2030	2020-2030	2020	2030	2020-2030
Global	11.76 (11.29 to 12.24)	14.25 (11.45 to 17.63)	21.17 (1.42 to 44.04)	2.98 (2.86 to 3.1)	3.44 (2.83 to 4.22)	15.44 (-1.05 to 36.13)
High SDI	20.16 (19.27 to 21.09)	19.52 (15.26 to 24.68)	-3.17 (-20.81 to 17.02)	2.36 (2.26 to 2.47)	2.33 (1.9 to 2.89)	-1.27 (-15.93 to 17)
High-middle SDI	27.96 (26.62 to 29.34)	30.88 (23.41 to 40.16)	10.44 (-12.06 to 36.88)	4.7 (4.48 to 4.92)	4.58 (3.23 to 6.43)	-2.55 (-27.9 to 30.69)
Middle SDI	9.69 (9.11 to 10.28)	13.58 (10.57 to 17.42)	40.14 (16.03 to 69.46)	2.42 (2.31 to 2.54)	2.83 (2.3 to 3.52)	16.94 (-0.43 to 38.58)
Low-middle SDI	8.11 (7.23 to 9.08)	9.8 (7.25 to 13.03)	20.84 (0.28 to 43.5)	2.77 (2.62 to 2.92)	2.94 (2.25 to 3.85)	6.14 (-14.12 to 31.85)
Low SDI	8.89 (8.59 to 9.19)	11.42 (9.23 to 14.02)	28.46 (7.45 to 52.56)	4.37 (4.21 to 4.54)	5.03 (4.05 to 6.21)	15.1 (-3.8 to 36.78)

SDI – socio-demographic index.
